# Laser wakefield accelerated electron beams and betatron radiation from multijet gas targets

**DOI:** 10.1038/s41598-020-73805-7

**Published:** 2020-10-08

**Authors:** Vidmantas Tomkus, Valdas Girdauskas, Juozas Dudutis, Paulius Gečys, Valdemar Stankevič, Gediminas Račiukaitis, Isabel Gallardo González, Diego Guénot, Jonas Björklund Svensson, Anders Persson, Olle Lundh

**Affiliations:** 1 Center for Physical Sciences and Technology, 02300 Vilnius, Lithuania; 2 Vytautas Magnus University, 44248 Kaunas, Lithuania; 3 Department of Physics, Lund University, 221 00 Lund, Sweden

**Keywords:** High-field lasers, Plasma-based accelerators, X-rays, Imaging and sensing

## Abstract

Laser Plasma Wakefield Accelerated (LWFA) electron beams and efficiency of betatron X-ray sources is studied using laser micromachined supersonic gas jet nozzle arrays. Separate sections of the target are used for the injection, acceleration and enhancement of electron oscillation. In this report, we present the results of LWFA and X-ray generation using dynamic gas density grid built by shock-waves of colliding jets. The experiment was done with the 40 TW, 35 fs laser at the Lund Laser Centre. Electron energies of 30–150 MeV and 1.0 × 10^8^–5.5 × 10^8^ photons per shot of betatron radiation have been measured. The implementation of the betatron source with separate regions of LWFA and plasma density grid raised the efficiency of X-ray generation and increased the number of photons per shot by a factor of 2–3 relative to a single-jet gas target source.

## Introduction

Laser Plasma Wakefield Accelerators (LWFA) are attractive alternatives to radio-frequency accelerators for a number of applications. LWFA enable the development of compact, next-generation X-ray and γ-ray sources for use in biomedical applications, physics, materials science, and significantly facilitate their access to universities and smaller research centres^1–3^. In LWFA an intense ultrashort laser is focused down to 10 µm into a gas jet. If the laser pulse reaches so called relativistic intensities around 10^18^ W/cm^2^, it will excite a non-linear plasma wave which accelerates electrons up to relativistic velocities. During their acceleration, the electrons oscillate transversally inside the plasma wave, which leads to the emission of X-ray radiation in the keV range defined as betatron emission. Additional work is, however, required to increase the energy and charge of accelerated electrons, and to reduce the energy dispersion of the electron beams. The charge and energy dispersion of accelerated electrons is controlled by tapered plasma channels using capillary discharge^4^, by ionization-assisted^5,6^ or self-truncated electron injection^7,8^, by injection utilising colliding beams^9,10^ or the density down-ramp triggered injection^11–13^. Density down-ramp injection is implemented by the formation of tailored plasma profiles using two or more stages of nozzles^14,15^ by insertion of a wire^16^, a razor blade^17^ or a capillary jet^18^ into the nozzle jet causing shock waves. Implementation of separate nozzles and insertion of external objects into the gas jet increases mechanical complexity, lowers the stability and is hard to realise in the case of laser pulses with duration < 10 fs where a dephasing length is less than 100 μm^19^. Tailored plasma channels are also interesting for optimisation of betatron radiation aiming to increase the critical energy, the number of photons per shot and brightness of X-ray and γ-ray sources. The efficiency of betatron radiation is raised by increasing the energy of electrons, plasma concentration and betatron radius. Tuning of the betatron radiation is implemented using two-stage nozzles: one with a lower plasma concentration, optimised for the acceleration of electrons, and the second one with a higher concentration – acting as a plasma radiator^20,21^.

In this report, we present the experimental results of LWFA and X-ray generation using the 40 TW, 35 fs laser at the Lund Laser Centre using novel multi-stage micronozzles manufactured from a single block of fused silica. The charge, energy and divergence of accelerated electrons and efficiency of betatron X-ray radiation were defined by the formation of gas density grid using shock wave interference. Fused silica nozzles are more resistant to optical damage than polymeric or metallic nozzles. Fused silica has by order of magnitude higher optical damage threshold^22^ comparing to polymeric structures^23^. The ablation threshold of fused silica^24^ is several times larger than for metals^25^. Furthermore, the softening temperature of the fused silica is larger than that of plastics and the melting point of metals, such as aluminium, copper or iron^26^. By repetitive use, the nozzles get damaged due to the plasma produced in the laser–gas interaction. For example, the gas flux of stainless-steel nozzles starts to show turbulence for less than 10^4^ shots due to nozzle damage^27^. The fused-silica nozzles demonstrated high resilience to damage and provided reliable and reproducible results after operation at kilohertz repetition rate for about 3–5 × 10^7^ shots^28^. Comparing to gas capillaries or gas cells, the supersonic nozzles targets are more easily to setup and can be combined to multijet longitudinal plasma profiles. The implementation of metallic nozzles can be more favourable in the conditions when the backing pressure exceeding 100 bar is required. The nozzles manufactured from fused silica are fragile and have to be handled with care. At the modest pressure of 20–60 bar fused silica nozzles ensure significantly longer operation times than metallic or polymeric nozzles. Laser-assisted hybrid microfabrication technique^29,30^ of fused silica allows the formation of centimetre-size frame and channels of the diameter less than 40 μm with the surface roughness < 1 μm from a single block. The formation quality exceeds the precision of 3D printing^31^ and electro-erosion technique^32^.

**Figure 1:**
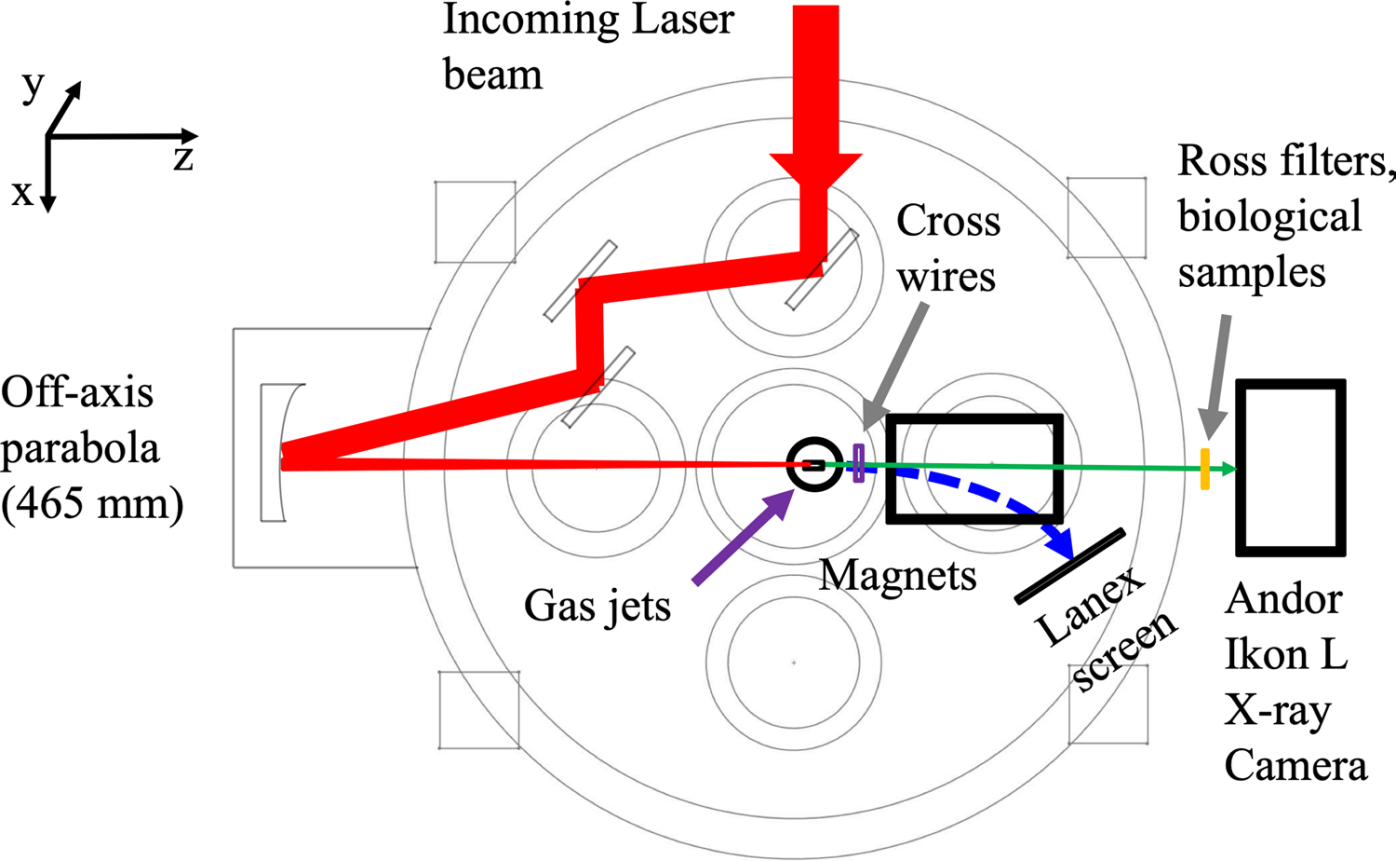
Experimental setup of the laser wakefield acceleration of electrons and measurement of betatron radiation.

**Figure 2:**
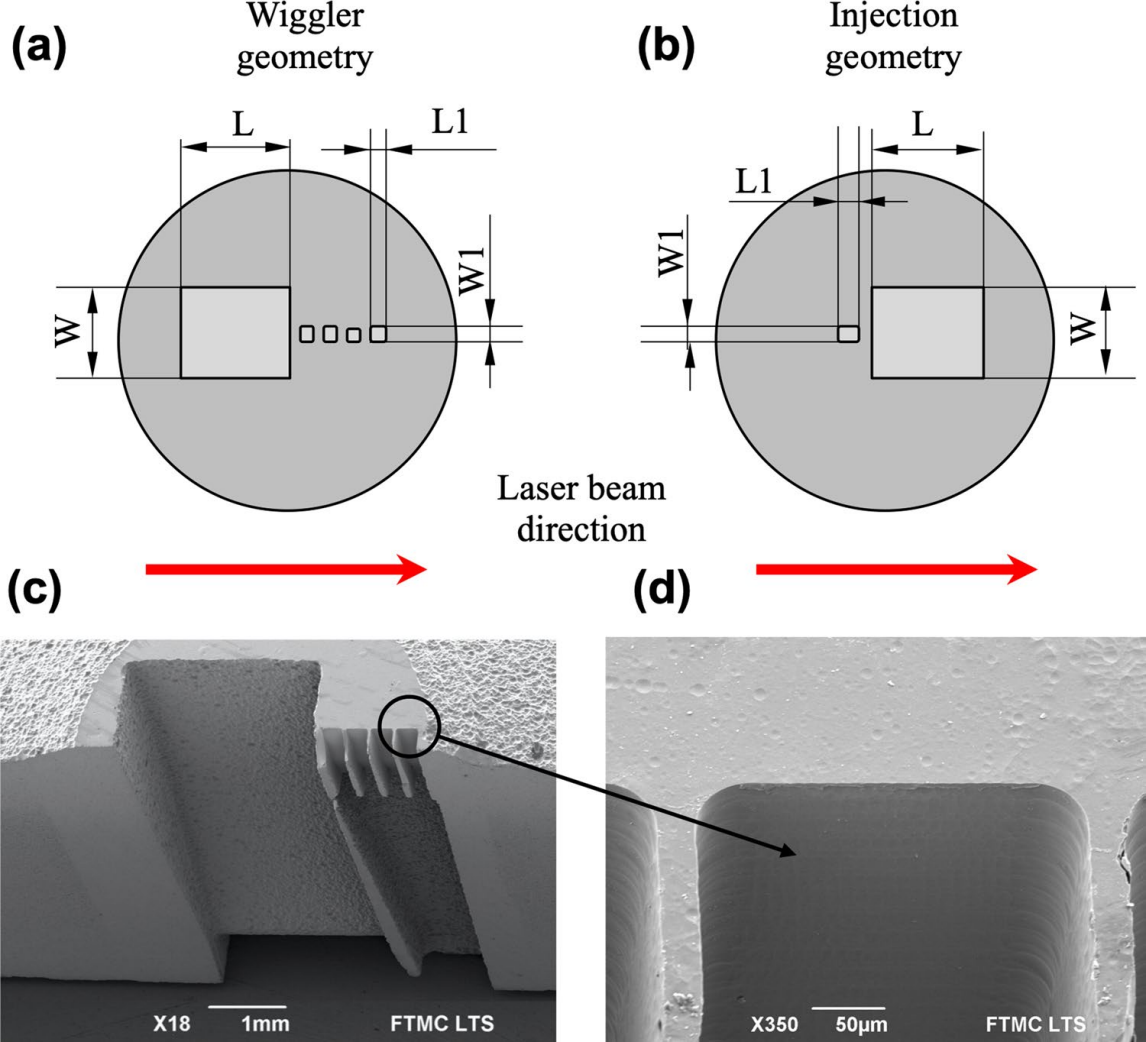
The geometry of the nozzle array **Arr2** in wiggler geometry (**a**) and **Arr4** in injector geometry (**b**). The red arrows indicate the orientation of the nozzle arrays relative to the incoming laser beam. SEM images of the cross-section of the array **Arr2** cut-in-half manufactured using the nanosecond rear side machining (**c**) and an element of the slit array with the length of 200 μm manufactured using the Femtosecond Laser-Induced Chemical Etching (FLICE) technique (**d**). **Arr2** consists of a converging–diverging 1.5 mm slit nozzle **S1** and four capillary nozzles with the fixed channel dimensions of 200 μm × 200 μm.

The experiment has been done using the experimental setup shown in Fig. 1 and LWFA parameters described in Table 2 in “Methods”. For the laser wakefield acceleration and X-ray generation, laser beam focused to the spot of 8 μm at FWHM, and plasma concentration 2 × 10^18^–1 × 10^19^ cm^−3^ were implemented. The peak laser intensity was 3.6 × 10^19^ W/cm^2^ and the corresponding laser strength parameter *a*_0_ = 4.0. The geometries and dimensions of the nozzle arrays used in the experiments are presented in Fig. 2a,b and in Table 3 (see “Methods”). The nozzle arrays were made from two sections. The first section comprises a main supersonic converging–diverging nozzle of 1.5 mm length (S1, Arr2, Arr4) or 2.25 mm (Arr3) length commonly used for laser-plasma acceleration. The second section consists of 1 or 4 smaller nozzles of 200–300 µm length located 100 µm away from the main nozzle (Arr2, Arr3, Arr4). The nozzles are oriented in wiggler or injector geometry. In wiggler geometry, the array is placed after the main nozzle, and in injector geometry, the array is placed before the main nozzle relative to the incoming laser beam. The SEM image of a cross-section of a nozzle array cut in half is shown in Fig. 2c,d.

The microarrays of gas-jet nozzles were designed by iterative numerical simulation of FBPIC PIC (Particle in Cell) and OpenFOAM algorithms. The energy and spatial distribution of the LWFA accelerated electrons were modelled using the FBPIC PIC simulation^33^. The obtained results were post-processed to evaluate the number of X-ray photons per shot and the brightness of electron-generated betatron radiation using the analytical expressions^34–36^ (see Supplementary information S1). The concentrations, velocities, pressures and temperatures of the gas jets were calculated using the OpenFOAM software^37^.

### Experimental results

**Figure 3:**
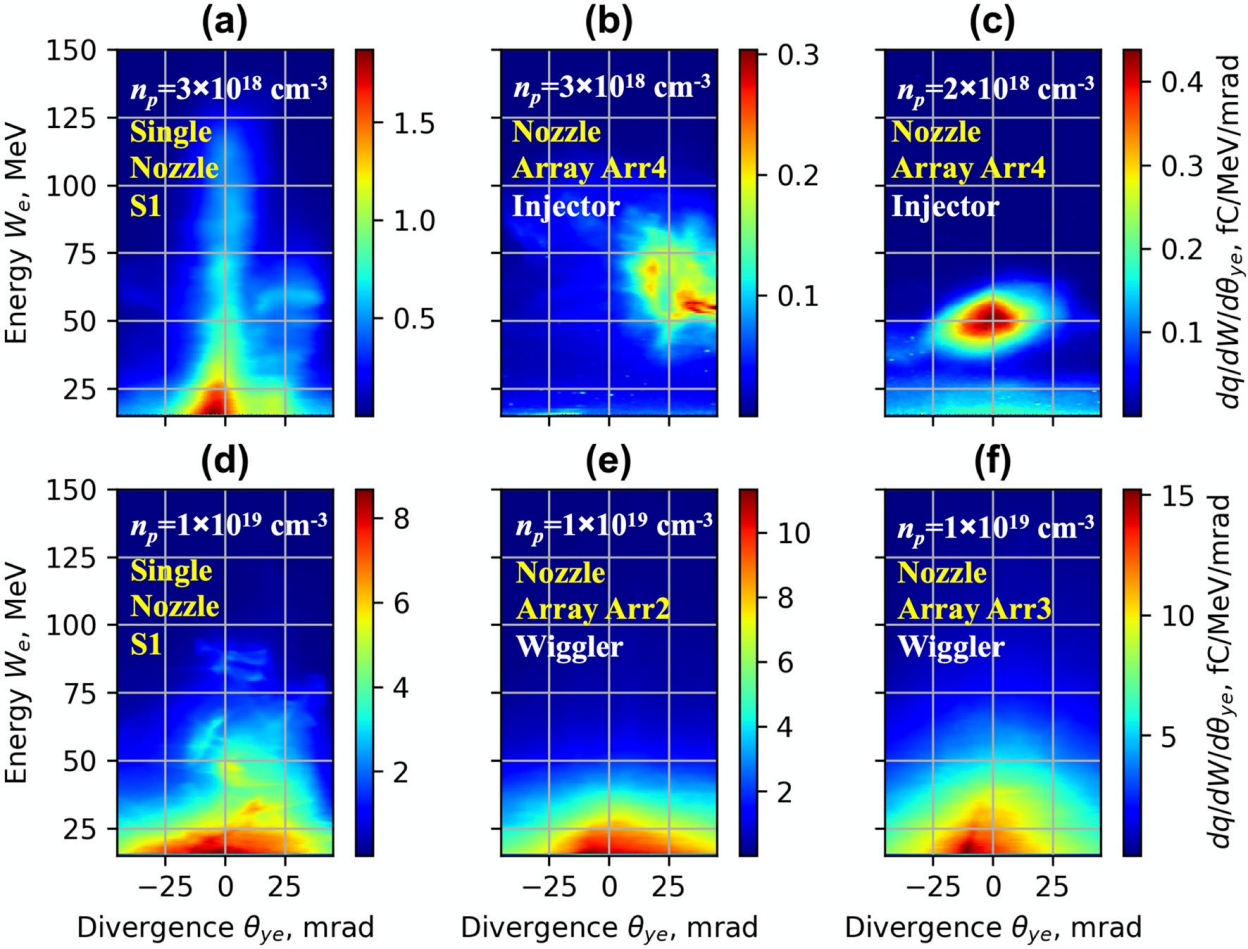
Experimental profiles of energy and spatial distribution of electrons for *n*_*p*_ = 3 × 10^18^ cm^−3^ for the single **S1** nozzle for the mixture He + 1% N_2_ (**a**) and the array **Arr4** in injector geometry for pure He (**b**) and *n*_*p*_ = 2 × 10^18^ cm^−3^ for the mixture He + 1%N_2_ (**c**). Experimental profiles of energy and spatial distribution of the charge of electrons for *n*_*p*_ = 1 × 10^19^ cm^−3^ for the single **S1** nozzle (**d**) and for the array **Arr2** (**e**) and **Arr3** (**f**) for the mixture He + 1%N_2_ in wiggler geometry.

The electron energy spectrum, divergence and charge of accelerated electrons were measured using a magnetic spectrometer and Lanex scintillation screen. The spectrum data and spatial distribution of charge of electrons averaged over 10 shots for a single S1 nozzle and Arr2, Arr3, Arr4 arrays are shown in Fig. 3. Highest energy and lowest divergence (FWHM) of accelerated electrons were obtained with a single nozzle and moderate focusing at a lower backing pressure of 5–12 bar of He + 1%N gas (Fig. 3a). Quasi-monoenergetic electron bunches were observed at a low backing pressure of 5–7 bar of pure He using an injector geometry of Arr4 with a density-triggered injection of electrons (Fig. 3b). The corresponding plasma concentration was approximately *n*_*p*_ = 3 × 10^18^ cm^−3^. The energy of separate electron bunches had low energy dispersion of 5–10 MeV. However, the amount of accelerated charge and spatial distribution fluctuated from shot to shot, which increased the averaged distribution. Using the mixture of He + 1%N and lowering the backing pressure by a factor of 1.5 the injector array enabled to get more stable quasi-monoenergetic electron bunches with a mean energy of 50 MeV and dispersion ± 10 MeV (Fig. 3c). Adding a wiggler array (Fig. 3e,f) as well increasing of the backing pressure up to 18–26 bar led to a massive injection of electrons and broadening of the beam. Note that, the wiggler geometry increased the efficiency of betatron radiation. In the case of (Fig. 3a,c–f), the spatial distribution of the charge and divergence of the electron beam of a single shot did not differ substantially from the average of 10 shots. The backing pressure of 18 bar for S1, Arr2 and 26 bar for Arr3 corresponded approximately to the plasma concentration of *n*_*p*_ = 1 × 10^19^ cm^−3^.

**Figure 4:**
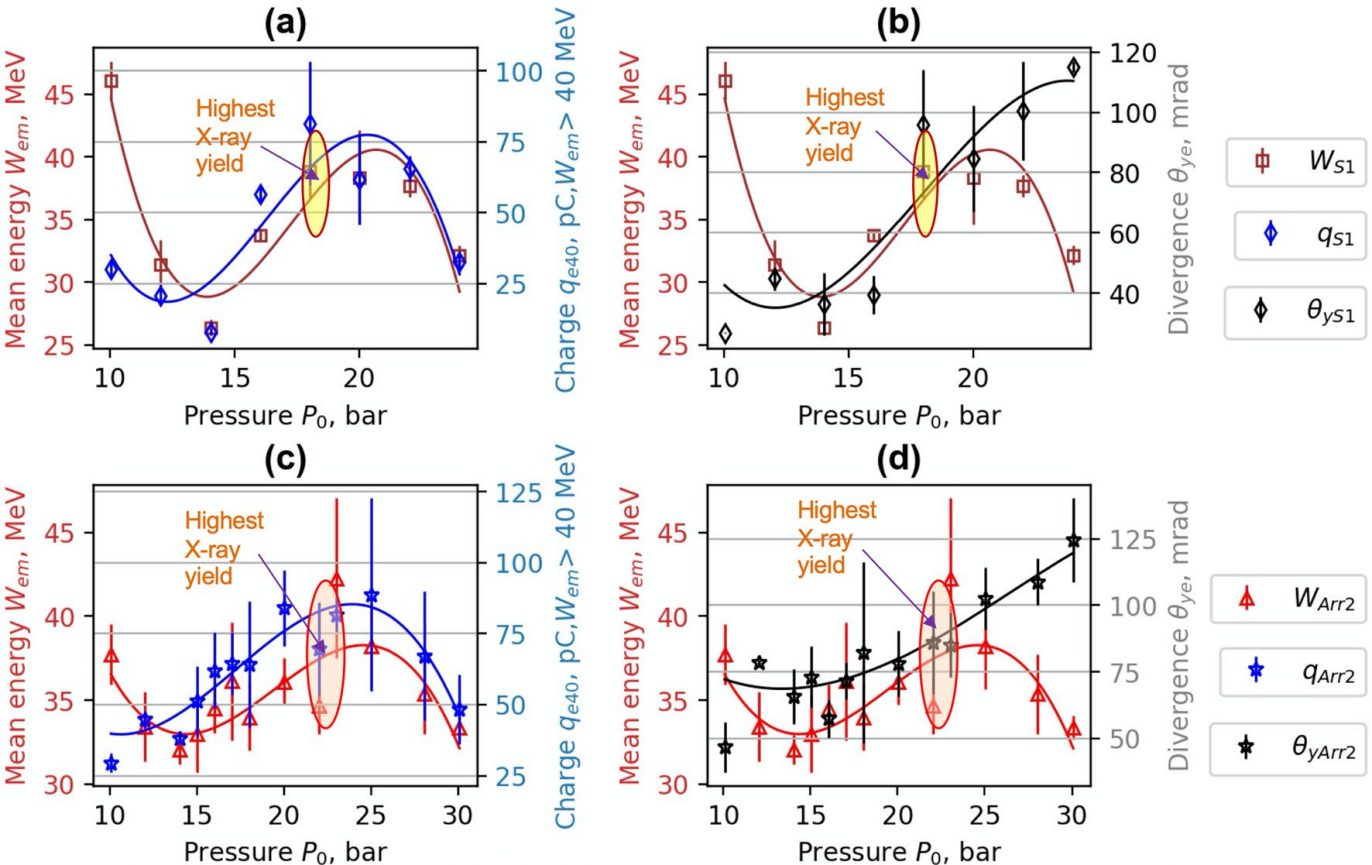
Experimental dependence of mean energy *W*_*mean*_, charge *q*_*40*_ for *W*_*mean*_ > 40 MeV (**a**,**c**) and mean divergence *θ*_*ye*_ (FWHM) (**b**,**d**) of accelerated electrons on gas backing pressure. ***W***_***S1***_*, q*_***S1***_*, ****θ***_***S1***_ correspond to the single nozzle **S1,** and ***W***_***Arr2***_,* q*_***Arr2***_, ***θ***_***Arr2***_ correspond to the array **Arr2** in wiggler geometry. Interpolated solid lines provide a guide to the eye.

**Figure 5:**
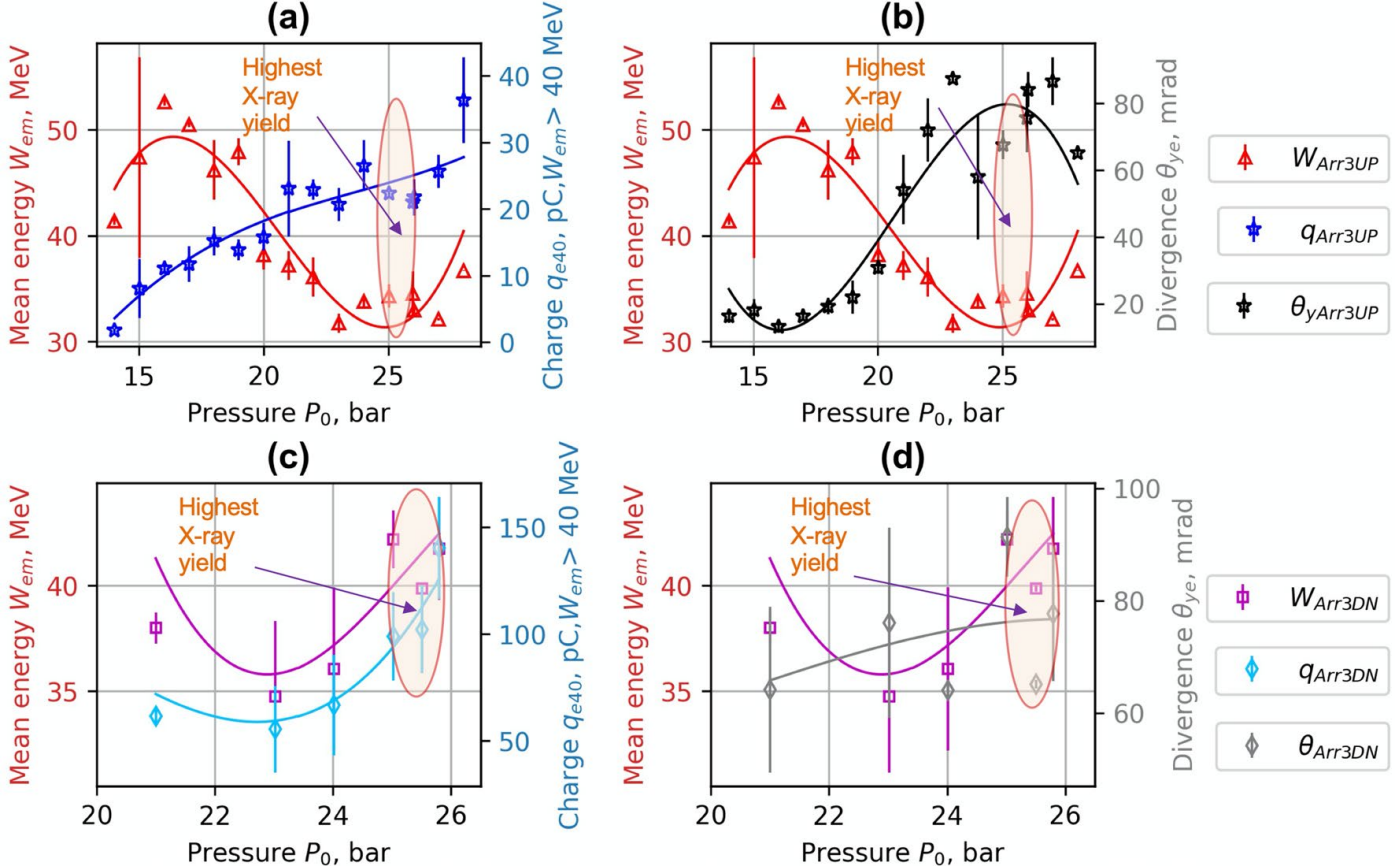
Experimental dependence of mean energy *W*_*mean*_, charge *q*_*40*_ for *W*_*mean*_ > 40 MeV (**a**,**c**) and mean divergence *θ*_*ye*_ (FWHM) (**b**,**d**) of accelerated electrons on gas backing pressure for the array **Arr3** in wiggler geometry. ***E***_***Arr3UP***_,* q*_***Arr3UP***_,* θ*_***Arr3UP***_ were measured at the distance of 800 μm, and—***E***_***Arr3DN***_,* q*_***Arr3DN***_,* θ*_***Arr3DN***_—at the distance of the laser beam above the nozzle of 400 μm. Interpolated solid lines provide a guide to the eye.

In Figs. 4 and 5, the dependence of mean energy, charge and divergence of accelerated electrons on gas backing pressure are presented. The arrows and the ellipses indicate the regions of the highest X- ray yield. For the 1.5 mm single nozzle S1, the maximum of X-ray radiation was reached at the backing pressure of 18–21 bar. (Fig. 4a,b). The maximum of X-ray counts was registered at the highest divergence values when the mean energy started to drop. At the lower backing pressure of 15 bar, adding the wiggler array to S1, increased the average divergence (FWHM) of accelerated electrons by 50% from 40 to 60 mrad. At the backing pressure of 20 bar, the average divergence of accelerated electrons for S1 and Arr2 was similar in the range of 70–90 mrad. However, for Arr2, the increase of the charge of accelerated electrons (*W*_*mean*_ > 40 MeV) and a drop of mean energy by 20% relative to S1 nozzle was observed.

The same trend of the increase of X-ray yield with the increase of divergence and charge was also observed for the array Arr3. The 2.25 mm cylindrical nozzle array Arr3 had longer acceleration distance by a factor 1.5 times relative to the nozzle S1 and array Arr2. The energy of accelerated electrons using this nozzle was higher. Arr3 nozzle had a higher expansion ratio than S1 and Arr2 nozzles. For the array Arr3, the maximum of X-ray radiation was reached at the backing pressure of 24–26 bar. At the distance of 800 μm of laser beam above the array Arr3 (Fig. 5a,b), when increasing the backing pressure from 15 until 28 bar, the charge grew continuously even though it remained low (in the range of 25–30 pC). In addition, the mean energy dropped from 50 to 32 MeV, and the divergence increased from 20 to 80 mrad. At the distance of 400 μm of laser beam above the array Arr3 (Fig. 5c,d), the substantial increase of charge (*W*_*mean* _> 40 MeV) up to 100 pC and mean energy to 43 eV was registered. The divergence of the electron beam remained at the same level of 80 mrad. Decrease of the distance between the array output and laser beam resulted in the growth of the charge by a factor of 4 and the enhancement of X-ray radiation by a factor of 5.

**Figure 6:**
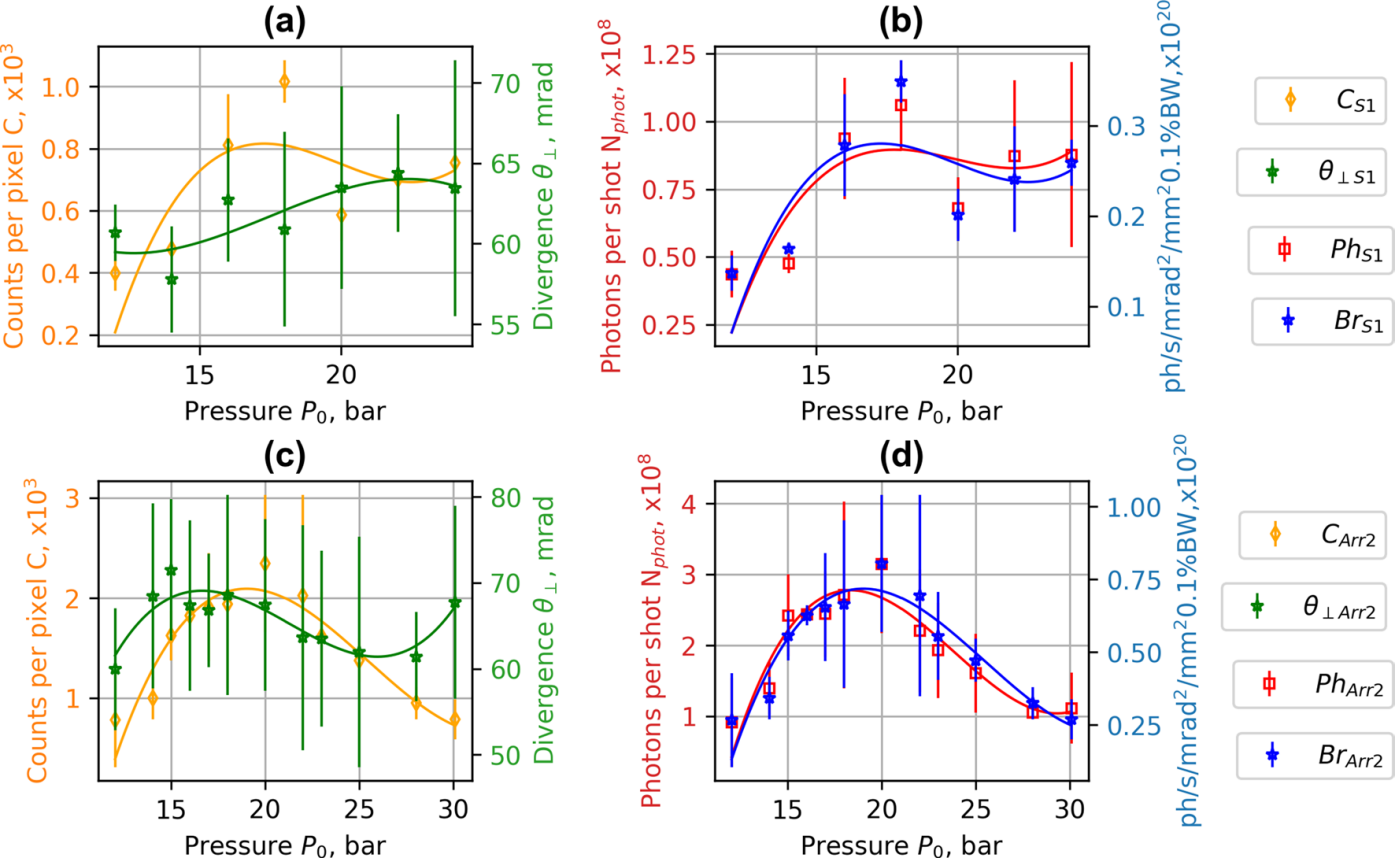
Experimental dependence of counts per pixel *C*, divergence *θ* (FHWM) (**a**,**c**), photons per shot *N*_*phot*_ and brightness *Br* (**b**,**d**) of X-ray radiation on gas backing pressure. ***C***_***S1***_, *θ*_***S1***_, *Ph*_***S1***_, ***Br***_***S1***_ correspond to the single nozzle **S1** and ***C***_***Arr2***_,* θ*_***Arr2***_, ***Ph***_***Arr2***_, ***Br***_***Arr2***_ correspond to the array **Arr2** in wiggler geometry. Interpolated solid lines provide a guide to the eye.

**Figure 7:**
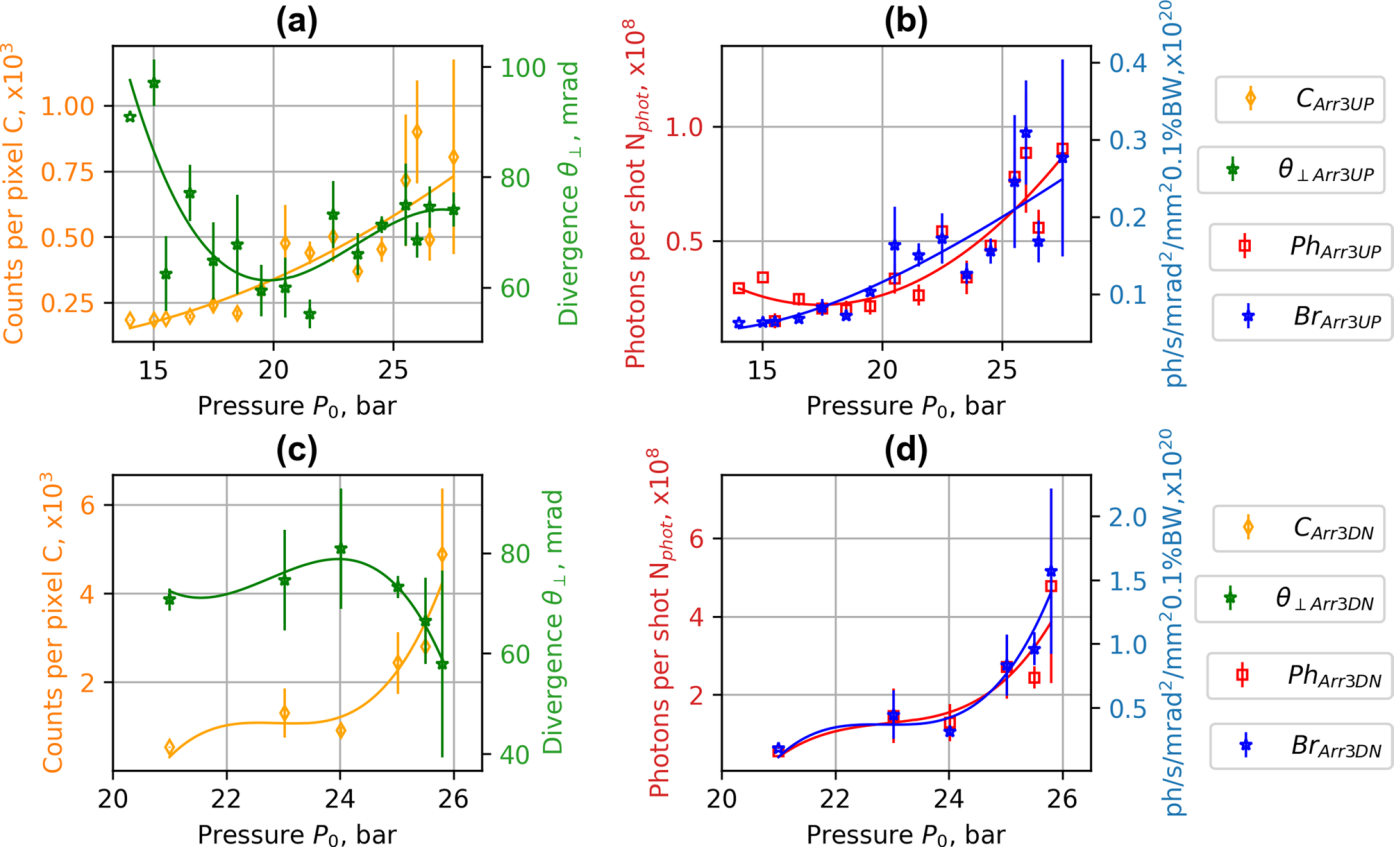
Experimental dependence of counts per pixel *C*, divergence *θ* (FHWM) (**a**,**c**), photons per shot *N*_*phot*_ and brightness *Br* (**b**,**d**) of X-ray radiation on gas backing pressure for the array **Arr3** in wiggler geometry. ***C***_***Arr3UP***_,* θ*_***Arr3UP***_, ***Ph***_***Arr3UP***_, ***Br***_***Arr3UP***_ were measured at the distance of 800 μm, and—***C***_***Arr3DN***_,* θ*_***Arr3DN***_, ***Ph***_***Arr3DN***_, ***Br***_***Arr3DN***_ at the distance of laser beam above the nozzle of 400 μm. Interpolated solid lines provide a guide to the eye.

**Figure 8:**
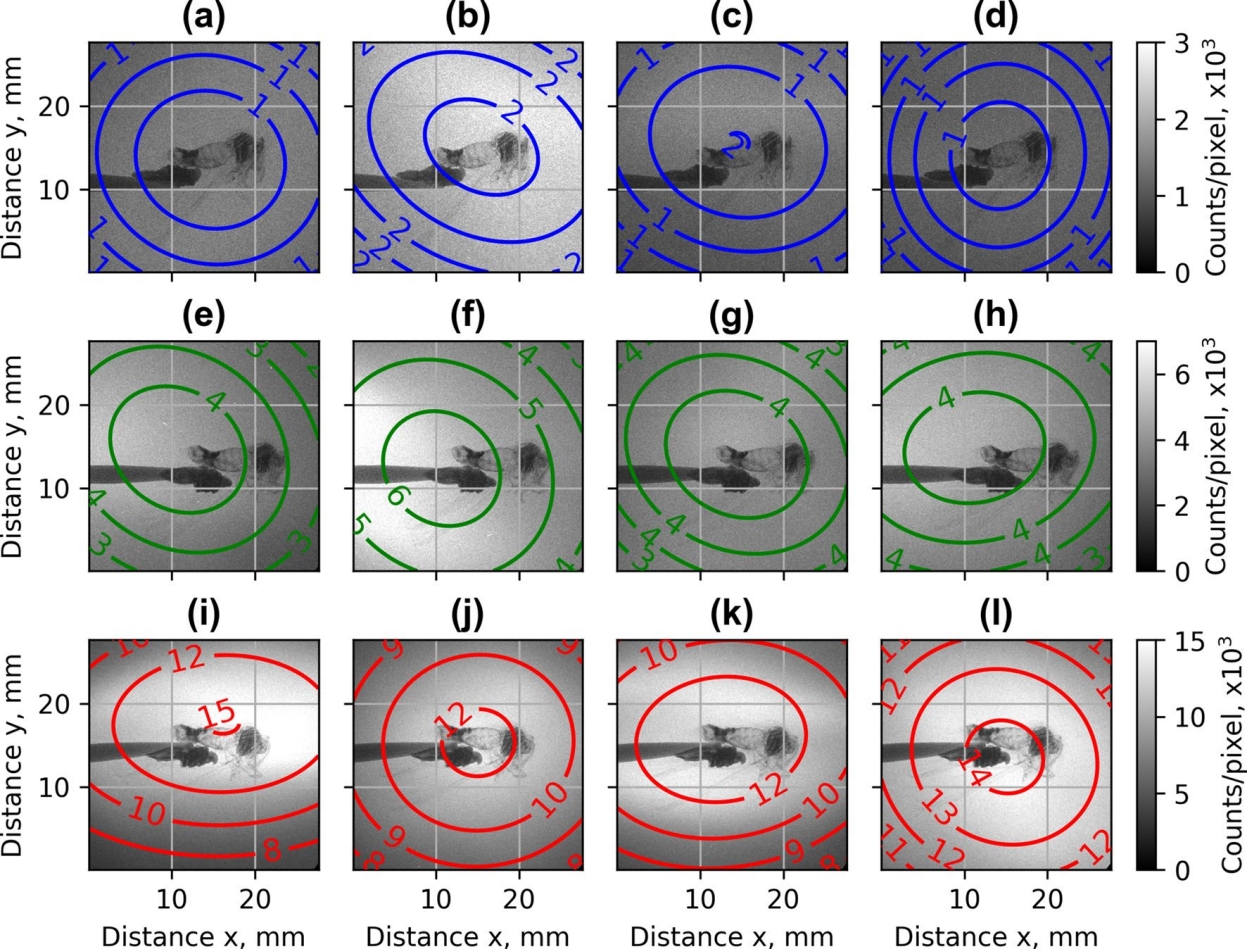
Examples of X-rays images, the colored ellipses represent regions with a number of counts per pixel higher than indicated (in thousands): single nozzle **S1** (**a**–**d**), array **Arr2 **(**e–h**), and array **Arr3** (**i–l**) at the distance of laser beam above the nozzle of 400 µm.

The experimental data of betatron X-ray radiation are presented in Figs. 6, 7, 8 and Table 1. The number of X-ray photons per shot and brightness increased proportionally to the amount of accelerated charge and the divergence of electron beam increasing the amplitude of betatron oscillation of electrons. The average energy of electrons with higher divergence is lower, however, and the maximum of X-ray radiation does not correspond to the maximum of the average energy of electrons. With increasing the pressure > 22 bar for S1 and Arr2 with the divergence of the electron beam increased further. The declining mean energy of electrons caused the drop in the number of photons of X-ray radiation. For the array Arr3, having a higher expansion rate of the gas jet, just the increase of X-ray radiation with the increase of pressure was observed. The highest number of photons and brightness of X-ray radiation was reached at 26 bar when the laser beam was propagating 400 μm above the nozzle. For the single S1 nozzle, and Arr3 at a laser-nozzle distance of 800 μm, the divergence of X-ray radiation (FHWM) increased proportionally to the divergence of the electron beam (Figs. 6a, 7a). For the array Arr2, and Arr3 at the laser-nozzle distance of 400 μm, the drop of the divergence of X-ray radiation (FHWM) by 15–30% was observed in the region of the backing pressure corresponding to the highest X-ray yield (Figs. 6c, 7c).

**Table 1:** The data of accelerated electrons and betatron radiation of a single nozzle and arrays in wiggler geometry for *n*_*p*_ = 1 × 10^19^ cm^−3^ and *a*_0_ = 4.0.

No.	Description	Charge > 40 MeV, pC	Counts per pixel, FHWM	Critical energy, keV	Diver-gence *θ* (*θ*_*ey*_*),* mrad, FHWM	Photons per shot, FHWM	Brightness, ph/s/mrad^2^/mm^2^/0.1%BW, FHWM
#1	1.5 mm single slit converging–diverging nozzle **S1**	70 ± 10	900 ± 300	2.3 ± 0.2	75 ± 10 (80 ± 20)	1.1 ± 0.5 × 10^8^	0.3 ± 0.1 × 10^20^
#2	1.5 mm slit converging–diverging nozzle with an array of 4 nozzles **Arr2**	85 ± 20	1800 ± 500	2.5 ± 0.2	80 ± 10 (80 ± 20)	2.9 ± 0.7 × 10^8^	0.7 ± 0.1 × 10^20^
#3	2.25 mm cylindrical converging–diverging nozzle with an array of 4 nozzles **Arr3** (distance 800 μm)	30 ± 10	900 ± 200	2.2 ± 0.2	50 ± 15 (80 ± 10)	1.0 ± 0.2 × 10^8^	0.3 ± 0.1 × 10^20^
#4	2.25 mm cylindrical converging–diverging nozzle with an array of 4 nozzles **Arr3** (distance 400 μm)	130 ± 25	4900 ± 1500	2.6 ± 0.1	70 ± 30 (90 ± 20)	5.5 ± 4.8 × 10^8^	1.6 ± 0.5 × 10^20^

In the #1–#3 cases, the laser beam was propagating 800 μm, in the #4 case—400 μm above the nozzle.

The field of view of the camera was only 35 mrad; the divergence values of X-ray radiation are extrapolated from the evaluated Gaussian distribution.

The critical energy of the betatron sources was measured using Ross filters. The spatial profiles of the betatron radiation were estimated using the relations^38–41^ of Gaussian approximation of intensity of X- ray radiation. The critical energy of betatron radiation was evaluated by calculating the squared difference between the measured transmission and the theoretical one for each critical energy of synchrotron radiation. The number of photons per shot was calculated by postprocessing the images at FWHM level of counts per pixel and corresponding critical energy *E*_c_.

The typical examples of X-rays radiation spatial distribution for the single nozzle S1 and arrays Arr2, Arr3 are shown in Fig. 8. The measurement of the divergence *θ* (FHWM) of X-ray radiation has demonstrated that the intensity distribution in the x–y direction changes from circular to elliptical from shot to shot. In average, the X-ray distribution for Arr3 was more elongated in x-direction parallel to laser polarisation relative to the X-ray distribution for S1 and Arr2 nozzles.

The radius size of betatron source was measured by imaging tungsten wires placed close to the betatron source, and comparison of diffraction intensity signals in the horizontal and vertical directions with the calculated Fresnel diffraction integrals for different X-ray source sizes^42–44^. The evaluation of Fresnel diffraction has shown that the radius of X-ray source changes from 3.5 ± 1 µm for single slit nozzle S1 and 11 bar of gas backing pressure to 7.5 ± 1 µm for array Arr2 and 29 bar of gas backing pressure. In the region of interest of measuring the brightness of X-ray corresponding to 15–26 bar of backing pressure, the source size was defined as 5 ± 1 µm in average in both horizontal and vertical direction. The fixed value of 5 µm was used for the brightness calculation of the source for all nozzles indicated in Table 1.

The spectra of betatron radiation of the single nozzle S1 and array Arr2 had the best match to the synchrotron radiation with the critical energy of 2.3 ± 0.2 keV and 2.5 ± 0.2 keV correspondingly. The spectrum of the array Arr3 for laser-nozzle distance of 400 μm had the best matching to the synchrotron radiation with the critical energy of 2.6  ± 0.1 keV. The divergence values *θ* (FWHM), presented in Table 1, were calculated by measuring separately and averaging of the ellipse radii afterwards. The numbers in parentheses in Table 1 indicate the mean divergence *θ*_*ye*_ of electrons in the y-direction. The implementation of the nozzle array Arr2 increased the critical betatron energy by 8–10% relative to the single nozzle S1. For the Arr2, the number of X-ray photons per shot relative to the single S3 nozzle increased from 1.1 × 10^8^ to 2.9 × 10^8^ and the brightness of the source from 0.3 × 10^20^ to 0.7 × 10^20 ^ph/s/mrad^2^/mm^2^/0.1%BW. For the array Arr3, the changing of the laser-nozzle distance from 800 μm to 400 μm increased the number of X-ray photons per shot from 1.0 × 10^8^ to 5.5 × 10^8^ and the brightness of the source—from 0.3 × 10^20^ to 1.6 × 10^20 ^ph/s/mrad^2^/mm^2^/0.1%BW.

**Figure 9:**
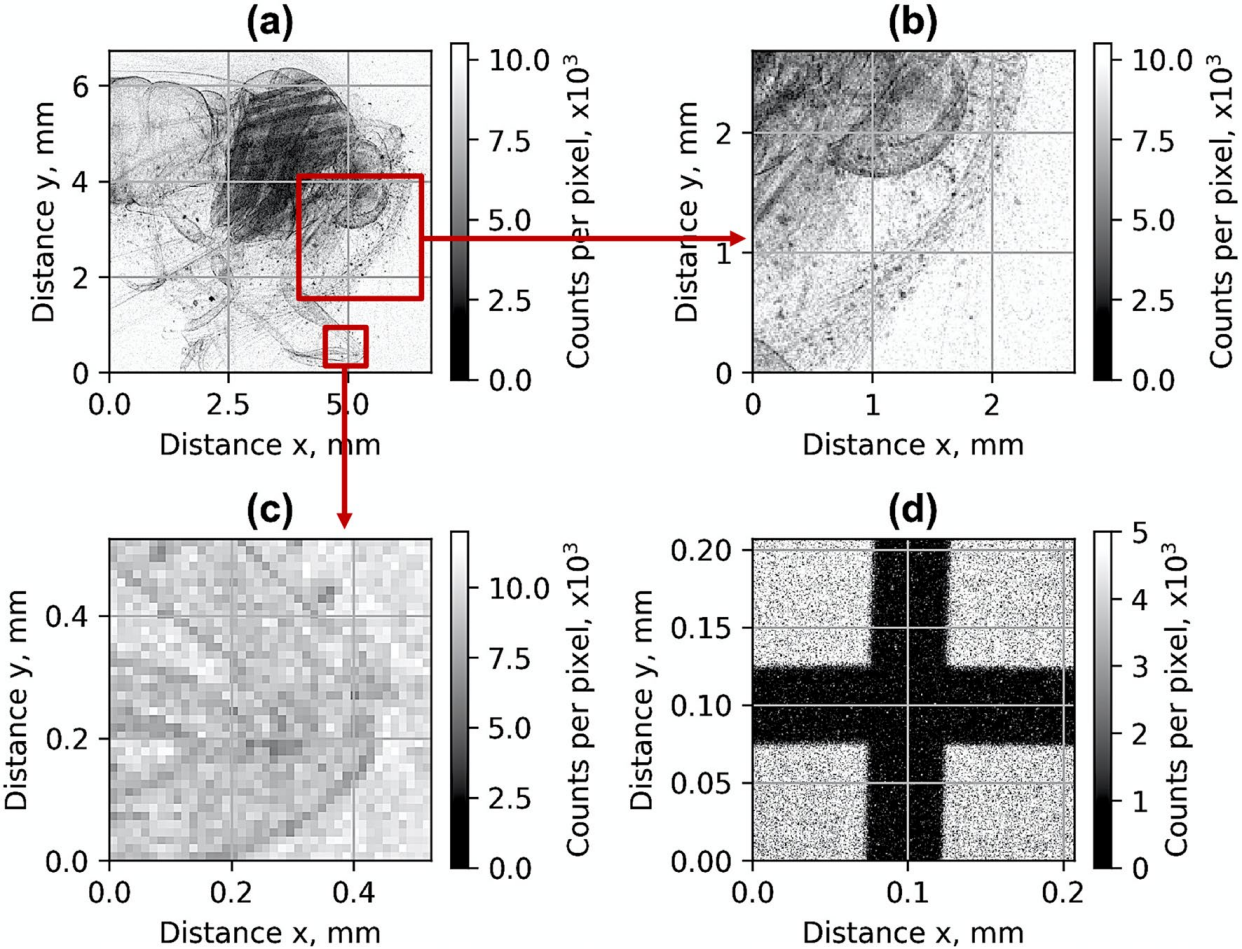
The X-ray transmission image (**a–c**) of a moth averaged over 10 shots and 50 μm tungsten cross-wires (**d**) for plasma concentration *n*_*p*_ = 1 × 10^19^ cm^−3^ using X-ray source of array **Arr3** for the laser beam propagating 400 μm above the nozzle.

The X-ray source was used for the transmission imaging of biological objects. In Fig. 9a–c, the X-ray images of a moth averaged over ten shots are shown. Small details as leg edges and hairs of the moth of approximatively 20 µm diameter can be seen (Fig. 9b,c). The moth was placed outside of the vacuum chamber at the distance of 6 cm before the X-ray detector. The magnification was 1.08, and the imaging resolution was limited by the pixel size of 13.5 μm of the detector. The X-ray source can be further implemented for phase-contrast imaging (PCI) and tomography^44^ allowing non-invasive analysis in low-absorbing materials, such as soft tissue. In-line, or propagation based, phase-contrast imaging (PB–PCI) requires no optical components. With appropriate propagation distances, the phase-shift evolves by itself into measurable intensity modulation. The PB-PCI imaging resolution was defined using the relations in reference^45,46^. In the case of Fig. 9d, the cross wires were located at 70 cm from the source. Assuming the Gaussian distribution, 10 μm diameter (FWHM) of the source, and magnification of 26, the imaging resolution was estimated to be 9 µm (defined as double the standard deviation of the source intensity distribution).

**Figure 10:**
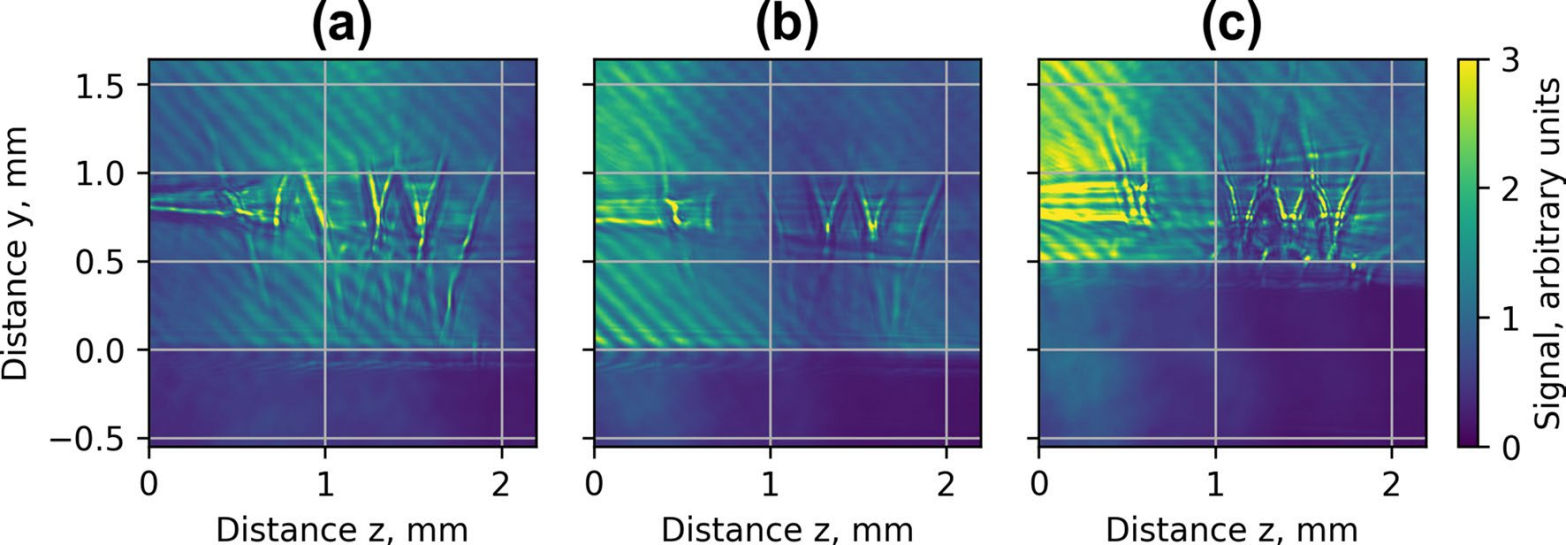
The interferograms of the array **Arr2** (**a**) and the array **Arr3** for the laser beam at the distance of 800 μm (**b**) and 400 μm (**c**) above the array for the *n*_*p*_ = 1 × 10^19^ cm^−3^.

The propagation of the laser beam and the formation of the plasma channel was monitored using a probe beam split from the driving laser and crossing the gas jet perpendicularly. The interferograms of Wollaston prism was used for shadowgraphy diagnostics. The interferogram images of array Arr2 and Arr3 (Fig. 10) showed the formation of shock waves of gas jets. For Arr2, the shock waves were observed at the laser-nozzle distance of 800 μm (Fig. 10a). For Arr3, the substantial difference in the distribution of plasma density depending on the distance of the laser beam above the nozzle is visible (Fig. 10b,c). At the distance of 800 μm (Fig. 10b), the width of the laser wakefield of the ionised region was narrower, in the range of 150–200 μm, and the shock waves only in the centre part of array Arr3 were visible. At the distance of 400 μm (Fig. 10c), the width of the ionised region increased to 200–250 μm, and the intersecting shock-wave fronts of all array nozzles were clearly visible.

### Numerical simulation

**Figure 11:**
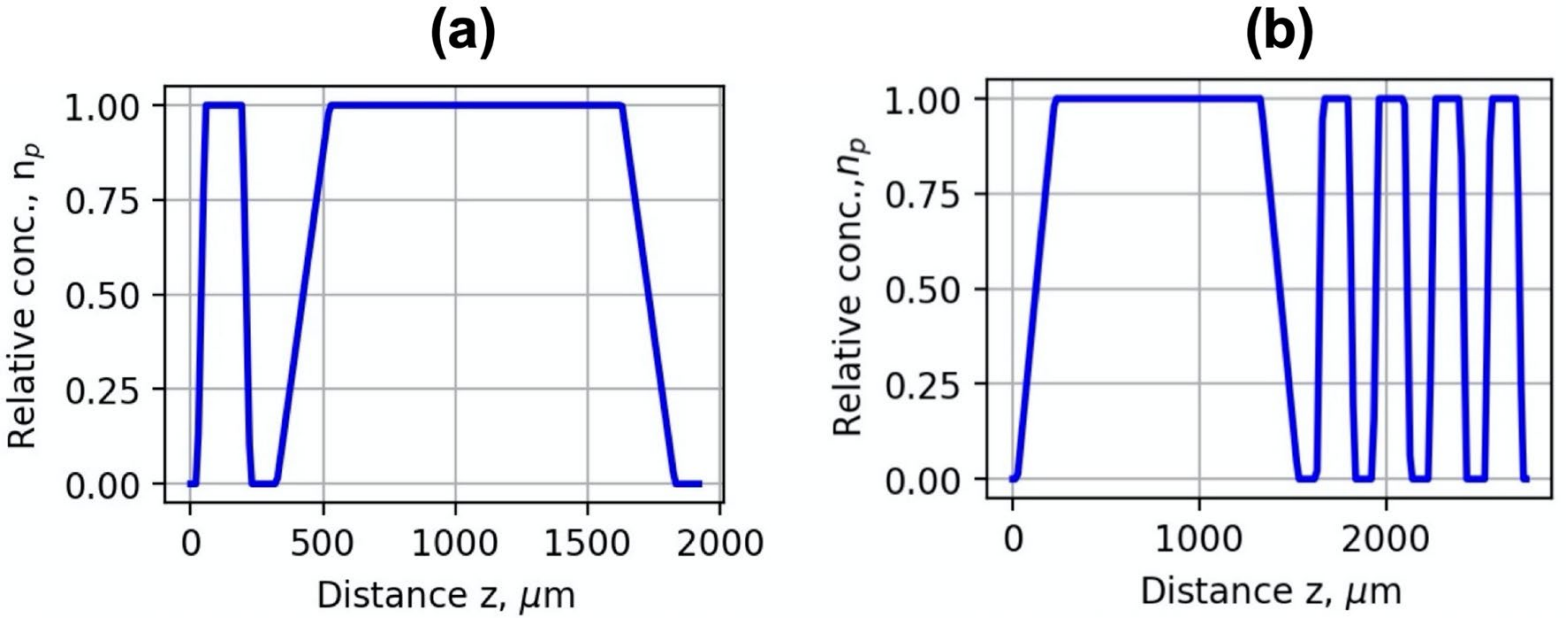
Simplified relative plasma density profiles of the injector (**a**) and wiggler (**b**) geometries used in the simulation.

To evaluate the impact of the plasma density grid on LWFA and X-ray radiation the iterative simulation process using the FBPIC PIC (Particle in Cell), and OpenFOAM algorithms was run. First, the simplified plasma concentration profiles were used in the FBIC simulation. The injector geometry was simulated by a 200 μm long plasma region, 100 μm gap and 1.5 mm plasma section for electron acceleration (Fig. 11a). A 1.5 mm long plasma acceleration section and wiggler comprising four 200 μm plasma regions and 100 μm gaps were used for the simulation of wiggler geometry (Fig. 11b).

Based on the FBIC LWFA results, several geometries of converging–diverging nozzles with different expansion rates were designed using OpenFOAM simulation, allowing to reach approximate plasma concentration profiles used in FBPIC simulation. The simulation parameters were chosen according to the experimental setup of Lund Ti:Sa laser. The laser strength parameter *a*_0_ was calculated, taking into account the focal length of the focusing mirror of 465 mm and the laser beam diameter at the FWHM level of 8 µm. It corresponded to the *a*_0_ = 4.0. The resolution of the mesh grid was 0.05 µm, and the time step was 0.17 fs. The simulation was performed for various acceleration distances, and the plasma concentration was changed within the range of 3 × 10^18^–1 × 10^19^ cm^−3^. Calculations were made for the gas mixture of He + 1%N_2_.

**Figure 12:**
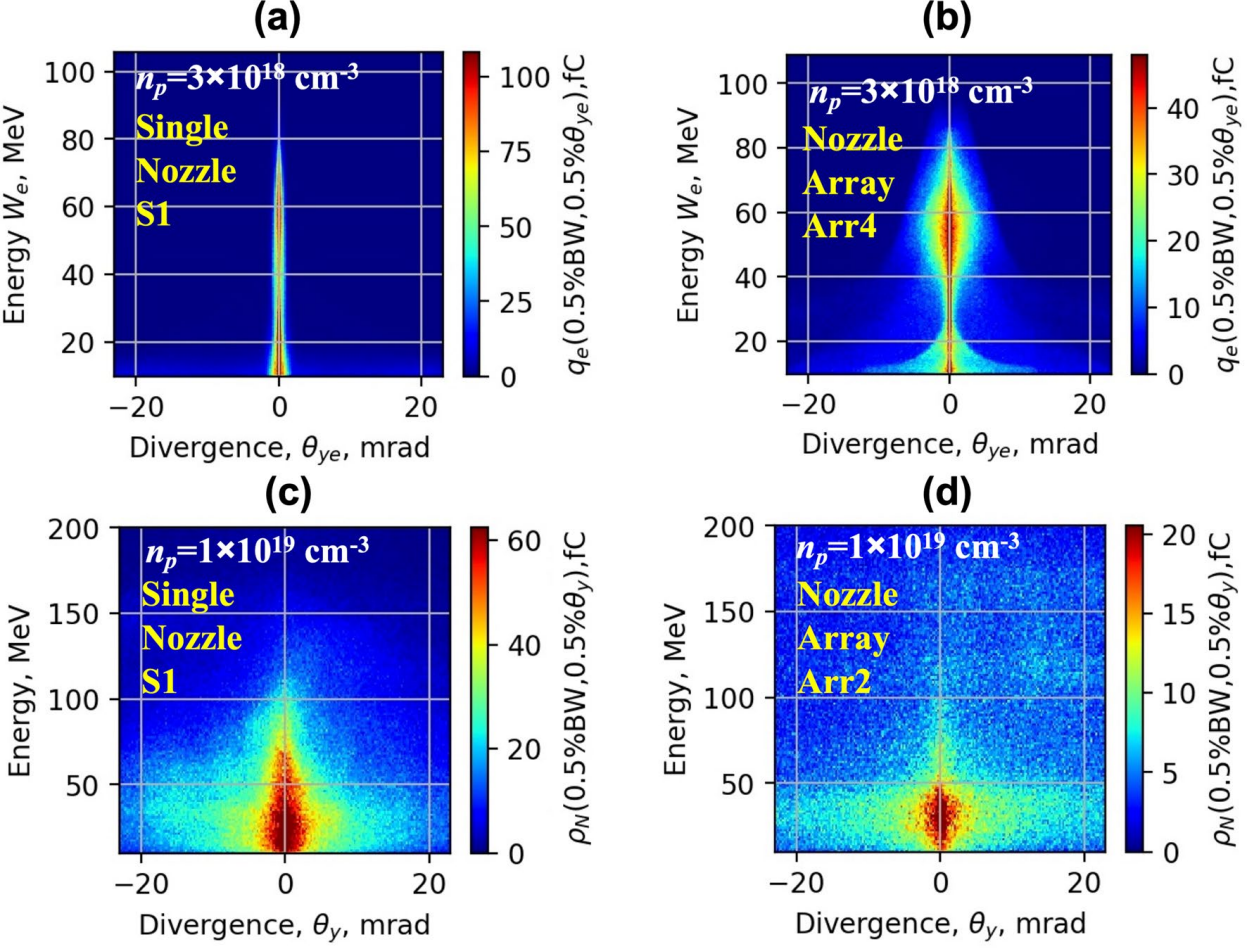
Simulated profiles of the transversal spatial distribution of the electron energy and charge for the single nozzle **S1** (**a**), array **Arr4** (**b**) in injector geometry for the mixture He + 1%N_2_, *a*_*0*_ = 4.0 and *n*_*p*_ = 3 × 10^18^ cm^−3^ at the acceleration distance z = 0.5 mm, and for *n*_*p*_ = 1 × 10^19^  cm^−3^ for the single nozzle **S1** (**c**) and array **Arr2** (**d**) at the acceleration distance z = 2.5 mm.

In Fig. 12a, the LWFA simulation results of the spatial distribution of energy and charge of ionization injected electrons for the single nozzle is presented. The matched plasma wavelength of *n*_p_ = 3 × 10^18^ cm^−3^ allows achieving high energy and the lowest divergence of accelerated electrons. The electrons are injected continuously, however, causing a broad energy spectrum of accelerated electrons. In Fig. 12b, the simulation results of the injection scheme using combined density-triggered and nitrogen ionization for Arr4 in injector geometry are presented. Adjusting the laser strength parameter and matching the plasma wavelength for *a*_0_ = 4.0 and *n*_p_ = 3 × 10^18^ cm^−3^ the formation of a quasi-monoenergetic bunch of higher charge was achieved.

In Fig. 12c,d, the simulation results of single nozzle S1 and array Arr2 in wiggler geometry at the plasma concentration *n*_*p*_ = 1 × 10^19^ cm^−3^ are shown. The energy of accelerated electron for a single S1 nozzle and *n*_*p*_ = 3 × 10^18^ cm^−3^ reaches 150–200 MeV, the divergence is 4–6 mrad. With the increase of the gas concentration from *n*_*p*_ = 3 × 10^18^ cm^−3^ to *n*_*p*_ = 1 × 10^19^ cm^−3^, the plasma wavelength becomes shorter and, several plasma waves are formed. The electrons are injected into the multiple plasma waves and the lateral plasma wake. The charge of the injected electrons increases, but the energy of accelerated electrons drops to 30–80 MeV, and the divergence of the electron beam increases to 20–40 mrad (Fig. 12c). In Fig. 12d, the simulation results of electron acceleration using the nozzle array Arr2 is presented. First, the electrons are accelerated in the gas jet of a longer section of array Arr2. Then, the electrons pass the second section, where the divergence of the electron beam increases up to 30–50 mrad.

**Figure 13:**
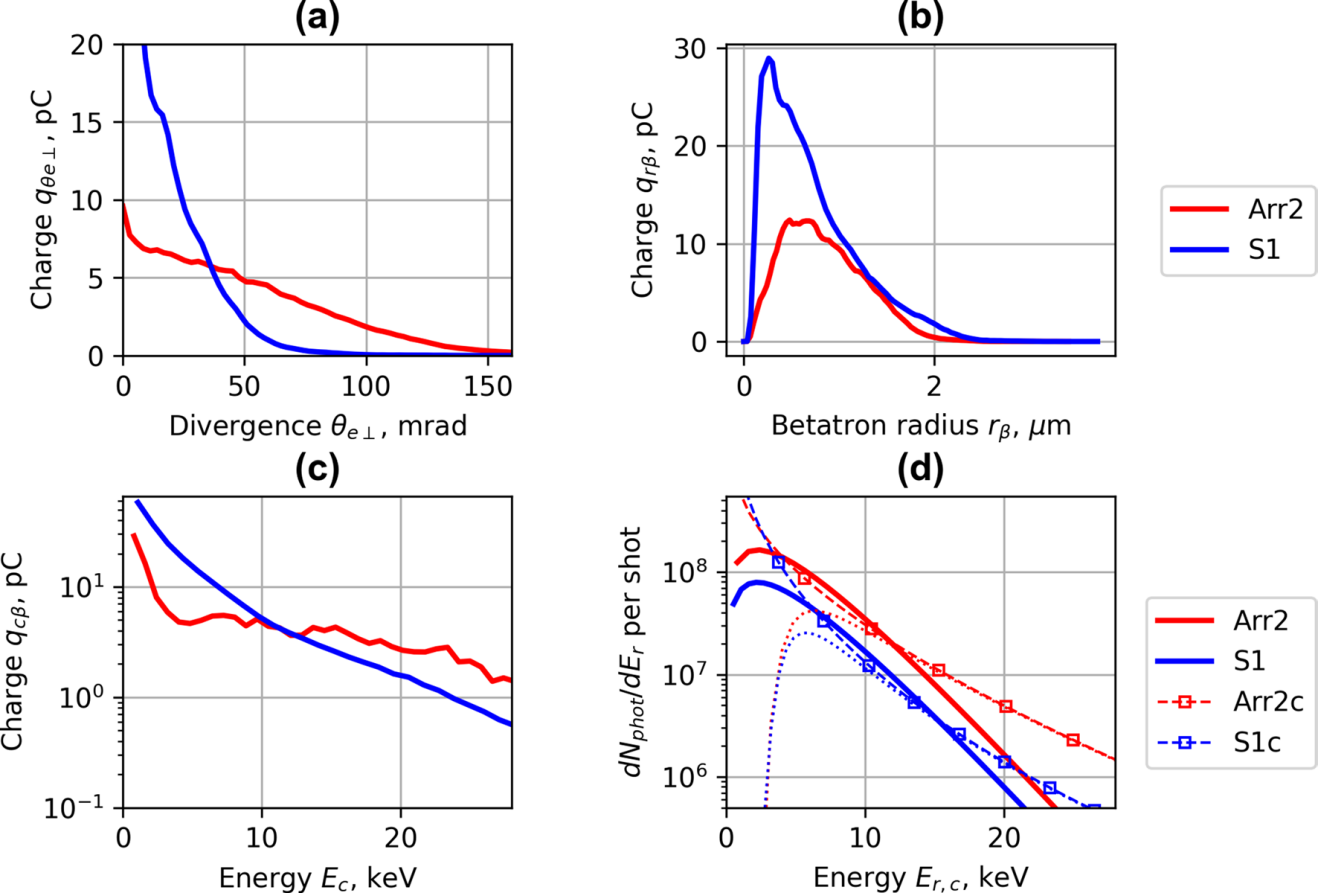
The simulated charge of electrons accelerated by array **Arr2** and single nozzle **S1** having divergence *θ*_*e*_ (FWHM) (**a**), betatron radius *r*_*β*_ (**b**), and critical synchrotron energy *E*_*c*_ (**c**). Calculated spectral density *dN*_*phot*_*/dE*_*r*_ of photon number per shot of electrons accelerated by array **Arr2** and single nozzle **S1** (**d**) for *a*_*0*_ = 4.0 and *n*_*p*_ = 1 × 10^19^  cm^−3^. The dashed lines **Arr2c** and **S1c** show the calculated density of *dN*_*phot*_*/dE*_*r*_, the dotted lines—the *dN*_*phot*_*/dE*_*r*_ attenuated by the materials placed between the X-ray source and camera, and the bold lines **Arr2** and **S1** show the best match of the synchrotron-like spectra with the mean critical energy *E*_*c*_ measured using Ross filters.

To estimate the parameters of X-ray betatron radiation, the data of momenta and spatial distribution of electrons simulation were post-processed using analytical relations (see Supplementary information S1 for details). In Fig. 13a–c, the amount of charge of electrons corresponding the divergence in the transversal direction *θe* (a), betatron radius *r*_*β*_ (b), and critical synchrotron energy *E*_*c*_ (c) are presented. The divergence in transversal direction *θe* (FWHM) was calculated as an average of the divergence of electrons in the *x*- and in *y*-direction. The betatron radius was defined as the transverse amplitude of electron motion in the ion channel produced in the wake of the laser pulse^47^.

The calculations have shown that the implementation of nozzle array Arr2 increased the number of electrons having a higher transversal divergence and slightly extended the average of betatron radius and critical energy. In Fig. 13d, the calculated spectral density *dN*_*phot*_*/dE*_*r*_ of radiated photon number per shot for the array Arr2 and single nozzle S1 is presented, where *E*_*r*_ is the energy of emitted photons.

The calculation demonstrated the increase of the number of photons per shot caused by the plasma density array by a factor of 2 and the total number of photon number reached 5 × 10^8^. The simulation results of critical energy *E*_*c*_ and the number of photons per shot were in good correspondence with experimental results. However, the calculated average radius of betatron *r*_*β*_ was in the range of 1–2 μm and differed from the size of 5 μm got by cross-wire diffraction measurements. It also resulted in mismatched values of the brightness of X-ray radiation.

**Figure 14:**
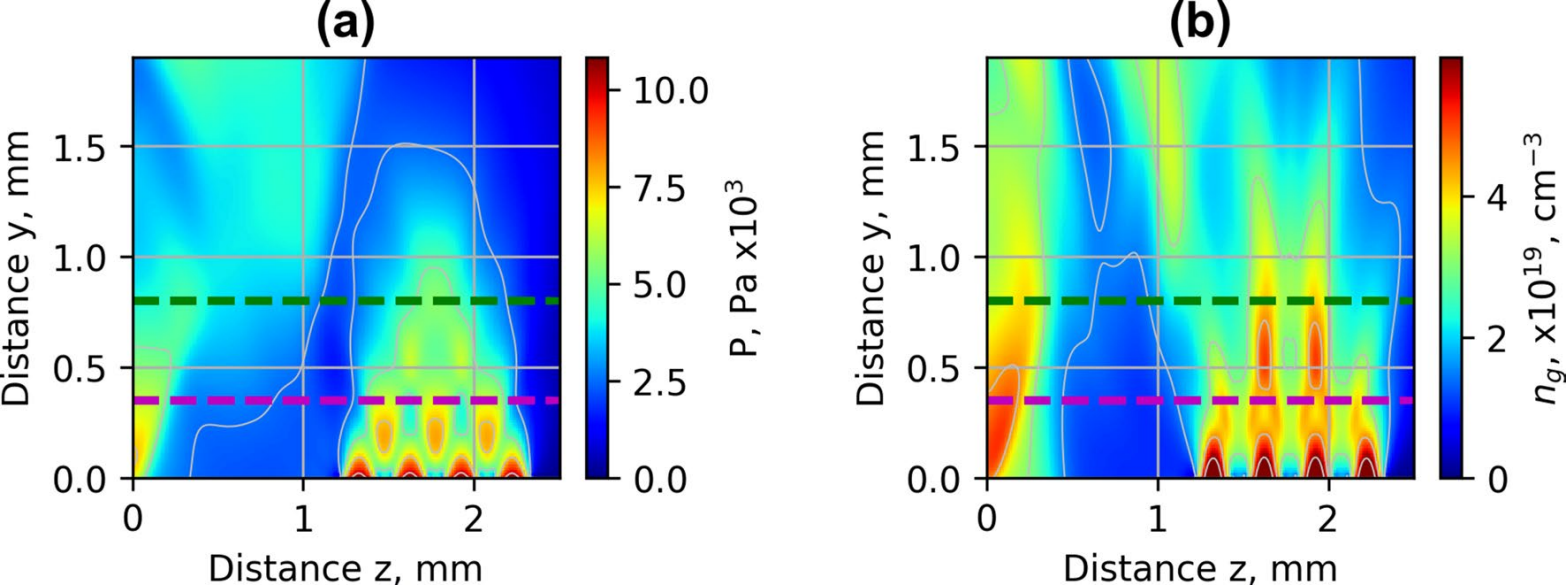
Simulated spatial distribution of pressure (**a**) and gas concentration (**b**) of He jet of **Arr3** at the backing pressure of 30 bar. The green dotted line indicates the distance of the laser beam of 800 μm, and the magenta dotted line shows the distance of 400 μm above the nozzle.

To interpret the experimental results of the spatial distribution of gas jets, the fluid simulation using the rhoSimpleFoam compressible stationary OpenFOAM solver was performed. In Fig. 14a,b, the simulation results of gas pressure and concentration at the backing pressure of 30 bar are presented. The simulation has shown the formation of diamond-shaped pressure (Fig. 14a) and density (Fig. 14b) grid is caused by the interference of shock waves of colliding jets. The shock waves also affected the region of the nozzle array used for LWFA. Thus, the region was compressed, and a shock wavefront between the first and second part of the array was built. The shock waves increased the gas density of the middle jets by a factor of 2 relatively to the edge jets without the neighbouring jet streams. The middle jets reached the height of 0.9–1.2 mm while the height of outside jets was 0.5–0.6 mm.

## Discussion

The data analysis of backing pressure scan has shown that the maximum of X-ray radiation corresponds to the maximum divergence of the electron beam while maintaining a high accelerated charge. For the single nozzle S1 and array Arr2 (Fig. 4), the maximum energy of accelerated electrons was reached at the backing pressure of 5–12 bar. At this backing pressure, the gas density matches the plasma bubble condition and is optimal for the acceleration of electrons. However, the divergence of the electron beam is small, and the yield of X-ray radiation is low. When increasing the gas density, even though the energy starts to drop, the accelerated charge grows, and the divergence increases a lot leading to an increase in X-ray flux. The maximal number of X-ray photons per shot was reached at a maximum of divergence and minimum of mean energy at the backing pressure of 18–21 bar for the single nozzle S1 and 24–26 bar for the array Arr2. The nozzle S1, Arr2 and Arr3 arrays have different gas expansion rates resulting in different gas concentration at the output of the nozzle. The corresponding gas concentration for S1 nozzle and Arr2 was reached at lower backing pressure relative to Arr3 nozzle. The distance of LWFA acceleration for Arr3 nozzle was longer than for S1 nozzle. Therefore, the energy of accelerated electrons and X-ray yield relative to S1 nozzle was higher. In wiggler geometry, the presented results of S1 and Arr2, Arr3 correspond to the highest photons per shot and brightness observed. In the injector geometry, the presented results of Arr4 correspond to the best results of the formation of quasi-monoenergetic electron bunches.

**Figure 15:**
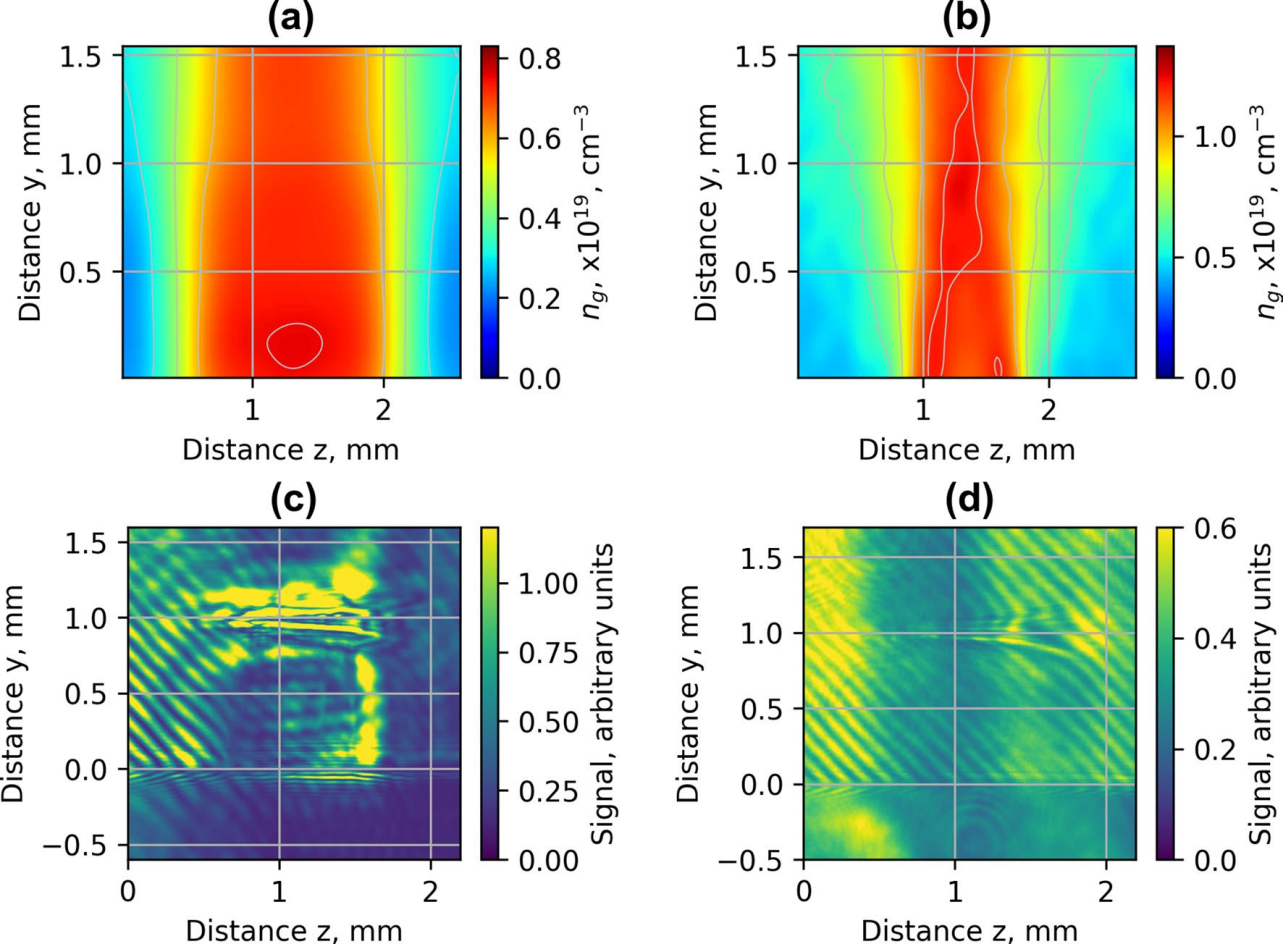
Gas concentration profiles reconstructed using measured wavefront sensor phase diagrams for the cylindrical converging–diverging nozzle with the diameter of 2.25 mm (**a**), slit converging–diverging nozzle with the length of 1.5 mm **S1** (**b**) at the backing pressure of 30 bar. Shadowgrams of the nozzle **S1** (**c**), and **Arr4** in injector geometry (**d**).

Implementation of nozzle arrays in wiggler geometry resulted in the increase of divergence of electron beam and growth of accelerated charge (Figs. 4, 5). The comparison of shadowgrams of plasma diagnostics of the S1, Arr2, Arr3 nozzles (Figs. 10, 15c) and the OpenFOAM simulation results (Fig. 14) confirmed the formation of the grid of maximums and minimums of gas concentration in the propagation path of laser beam approximately corresponding to the plasma density profiles simulated using FBPIC algorithm. The modelled accelerator and wiggler plasma sections indicated the growth of divergence of accelerated electrons and photon number of X-ray radiation relative to a single plasma section profile. For the array Arr2, the shock waves were present at the laser-nozzle distance of 800 µm. For the Arr3 having higher gas jet expansion rate, the laser beam did not cross all the intersecting jets of smaller nozzles of the diameter of 200 μm at the height of 800 from the output of nozzles. Hence, only by changing the distance between the laser beam and nozzle output from 800 to 400 µm, the laser beam propagated through the whole region of varying gas density. The gas density grid increased the transversal motion of electrons and increased the efficiency of X-ray radiation. The spatial distribution of X-ray radiation varied from circular to elliptical one as well as the orientation of elliptical distribution altered from shot to shot. It indicated that the divergence and orientation of X-ray radiation depend on the spatial distribution of plasma rather than on laser polarization. In average, the X-ray distribution was more elongated in x-direction parallel to laser polarisation, however. The interference of shock waves is defined by the period of nozzle array as well as by the pressure, temperature, and expansion rate of colliding jets. The numerical plasma simulation has shown that using array in wiggler geometry increases the divergence of accelerated electrons (Figs. 12c,d, 13a) by 30–50%. More electrons have higher critical energy of synchrotron radiation, and their contribution to the total X-ray emission is more extensive (Fig. 13c,d). The ratio of transversal and longitudinal momenta of electrons and betatron radius changes at a smaller extent in the range of 1–2 μm (Fig. 13b). The radius of the betatron source measured experimentally was 5 ± 1 μm on average. The difference between the calculated and experimentally measured radius of X-ray source could be explained by a much broader divergence of electrons corresponding to the transversal distribution radius > 50 μm at the acceleration distance of 2.5 mm. The resulting spectrum and source size of X-ray radiation is defined by the superposition of radiation of electrons with diverse momenta radiating at different spatial positions. We assume the main contribution to the X-ray radiation is from electrons of higher energy concentrated in the radius of 5 µm approximately. The X-ray radiation from the central 5 µm radius region can also explain the decrease of X-ray divergence (FWHM) observed at the backing pressure corresponding to the highest X-ray radiation yield. The electrons located in the broad halo of 50 µm have lower energy and do not contribute substantially to the X-ray radiation with the photon energy of several keV.

## Conclusion

In conclusion, the implementation of the nozzle array with separate regions of LWFA and gas density grid raised the efficiency of X-ray generation and increased the number of photons per shot by a factor of 2–3 relative to a single-jet gas target source. The critical energy of synchrotron radiation measured using Ross filters was in the range of 2.2–2.6 keV. The radius of the X-ray source in the region of the best X- ray yield was defined as 5 µm on average. The number of photons per shot was in the range of 1.0 × 10^8^–5.5 × 10^8^, and the brightness was 0.3 × 10^20^–1.6 × 10^20^ ph/s/mrad^2^/mm^2^/0.1%BW. The analysis of experimental data and numerical simulation has shown that the increased efficiency of X-ray radiation was caused by the formation of a gas density grid created by colliding jets. The experimental results of the energy and number of photons per shot were in good correspondence with the results of FBPIC simplified model. The calculated betatron radius of 1–2 μm differed from the measured X-ray source size of 5 µm however. The difference in sizes can be explained by the spatial distribution of radiating electrons in the radius of 50 µm on average. Also, the fixed betatron radius used in the definition of brightness has added the uncertainty in the calculations. The imaging tests of the biological samples in the transmission X-rays demonstrated the resolution higher than 20 μm. The resolution was limited by the pixel size of the detector of 13.5 μm. The X-ray source can be implemented for phase-contrast imaging and tomography. The estimated PB-PCI source size-limited resolution was 9 μm. In future work, it would be advantageous to test the nozzle arrays with independent adjustment of backing pressure of gas supply of accelerating and wiggler section. It would allow maximizing the energy of accelerated electrons and divergence of the electron beam. The collected data will be used for the optimisation of micronozzle geometry and efficiency of the X-ray source. The laser-assisted fabrication technology of the micronozzles from single fused silica block ensures higher precision, resistance to optical damage, and the formation of tailored plasma profiles with the dimensions of less than 40 µm with surface roughness < 1 μm.

## Methods

### Experimental setup

The diagram of the experimental setup for LWFA of electrons and betatron radiation measurement is presented in Fig. 1. In Table 2, the LWFA parameters in the wiggler and injector geometry are shown. For the LWFA experiments, the 800 nm 1.8 J Ti:Sa laser at the repetition rate < 1 Hz with a pulse duration of 35 fs, and pulse energy on the target of 700–880 mJ was used. The laser beam was focused on the gas jet in a vacuum chamber using a 4-inch offset parabolic mirror with the focal length of 465 mm, leading to a focal spot of 8 µm at FWHM. The laser strength parameter *a*_0_ was defined as the maximum amplitude of the normalized vector potential of the laser field *A*: *a*_0_ = *eA*/*m*_e_*c*^2^, where *A* is the maximum amplitude of the laser electric field, *e*—electron charge, *m*_e_—electron mass, and *c* is the speed of light in vacuum^1^. In practical units, the *a*_0_ parameter for the linearly polarized laser field with a Gaussian radial profile was calculated as *a*_0_^2^ = 7.3 × 10^−19^ [λ_L_(μm)]^2^ *I*_0_(W/cm^2^), where *λ*_L_ – is the laser wavelength and *I*_0_ – is the laser intensity. The aberration correction of the laser beam has been controlled by a deformable mirror, which ensured the high quality of the focused beam. The laser polarisation was horizontal along the x-axis. The energy of the accelerated electrons was measured using a Kodak Lanex Regular scintillation screen with a dipolar 0.8 T magnet of the total length of 20 cm located at the distance of 50 mm from the source. The width of the magnetic spectrometer was 5 cm, and the gap of the dipole gap was 15 mm. The measuring range of the spectrometer was 20–300 MeV. The relation between electron energy and position on the scintillating screen were determined by simulation using COMSOL Multiphysics and the measured map of the magnetic field. The energy resolution at 300 MeV was 1.3%/mrad. The uncertainty introduced by the ± 10 mrad divergence of the 125 MeV electron beam was 7.5 MeV, corresponding to the accuracy of ± 6%.

**Table 2:** The experimental LWFA parameters.

	Geometry	Laser strength parameter *a*_0_	Plasma concentration, cm^−3^	Laser spot FWHM, μm	Gas
1	Injector	4.0	3 × 10^18^	8	He
2	Injector	4.0	2 × 10^18^	8	He + 1%N_2_
3	Wiggler	4.0	1 × 10^19^	8	He + 1%N_2_

Scintillation screen images were captured using the Andor Zyla 4.2 Plus CMOS 16-bit camera. The camera was of 2048 × 2048 pixels with a pixel size of 6.5 μm. The energy and divergence of the electron beam were calculated using the images of Lanex scintillation screen. The response of the full imaging system was calibrated using previously published calibration factors of 6.95 × 10^–8^ pC/count for the scintillating screens measured at linear accelerator^48,49^. The betatron radiation was registered using the Andor iKon-L SO X-ray camera, sensitive to X-rays up to 20 keV. The X-ray CCD was of 2048 × 2048 square pixels with a pixel size of 13.5 μm. The camera window was made of 250 μm-thick beryllium. A 50 μm Kapton window with an additional shield of 3 μm aluminium foil was installed at the outlet of the vacuum chamber. The X-ray camera was installed at a distance of 73 cm from the source, and the betatron radiation passed through the air gap of 12 cm. To characterize the flux distribution and energy distribution of the X-ray pulse, a Ross-filter array^50^ was inserted into the X-ray beam at the distance of 6 cm from the exit of the experimental chamber. The filter array used was composed of intersecting strips of the elements Ni, Cu, Zn, Al, Ti, Zr, Cu of thickness 5 μm, 25 μm, 5 μm, 6 μm, 6 μm, 3 μm, 3 μm respectively. The transmission of the betatron X-rays through the filters was measured using Andor iKon-L Xray camera and compared to the transmission of synchrotron-like spectra with critical energy *E*_c_ using least-squares fitting method^51^. The radius of the betatron source was determined by measuring the Fresnel diffraction from the 50 μm tungsten cross-wires, placed 28 mm from the source, magnifying the image 26 times and comparing the intensities of X-ray signals in the horizontal and vertical directions. The intensity distribution of the Fresnel diffraction was calculated using the relations given in^42–44^.

### Nozzle manufacturing

The geometry and dimensions of nozzle arrays are presented in Fig. 2 and Table 3. The designed micronozzles were manufactured from fused silica with a thickness of 6.3 mm, using the second harmonic radiation (532 nm) of the DPSS nanosecond laser (from Ekspla) and the second harmonic radiation (515 nm) of the femtosecond Yb:KGW Pharos laser (from Light Conversion).

**Table 3:** The list of nozzles used in the experiment.

No.	First section			Second section		
	Description	Output dimensions, mm (L × W)	Throat dimensions, mm, (L2 × W2)	Description	Output dimensions, mm, (L1 × W1)	Throat dimensions, mm, (L3 × W3)
#1	Single slit converging–diverging nozzle **S1**	1.5 × 1.2	1.5 × 0.12	–	–	–
#2	Single slit converging–diverging nozzle **Arr2**	1.5 × 1.2	1.5 × 0.12	Slit array of 4 nozzles	0.2 × 0.2	0.2 × 0.2
#3	Single cylindrical converging–diverging nozzle **Arr3**	Φ2.25	Φ0.5	Slit array of 4 nozzles	0.2 × 0.2	0.2 × 0.2
#4	Single slit converging–diverging nozzle **Arr4**	1.5 × 1.2	1.5 × 0.12	Single slit converging–diverging nozzle	0.3 × 0.27	0.3 × 0.18

All nozzle frames were fabricated using the nanosecond laser and a rear-side or bottom-up machining approach alone, which allowed a high material removal rate^29,52^. However, depending on the design, some of the inner channels were fabricated in combination with the front-side laser ablation. This step was required due to the incapability of the rear-side machining technique to fabricate high negative taper angles, which are unavoidable in the configuration of converging–diverging Laval nozzles. Additionally, the output surface of nozzles with small channels was mechanically polished to reduce the gas concentration drop due to the chamfered edges^30^. For the formation of structures smaller than 200 μm, the femtosecond laser-induced selective chemical etching FLICE technique was implemented. The FLICE technique ensured the formation of microchannels with the surface roughness less than 1 μm^31,52,53^.

### Nozzle characterisation

Characterisation of gas density profiles of the nozzle used in the LWFA experiments was performed using an optical wavefront sensor, the collimated light beam of a HeNe laser, and the Python library of the open-source 3D tomographic image reconstruction algorithms “Astra Toolbox”^54^. The integral phase change was recorded by the wavefront sensor in two orthogonal planes perpendicular to the nozzle symmetry axis. The wavefront sensor resolution was 157 × 117 pixels with a pixel size of 17 µm. Using the integral phase distribution *φ*(x,y,z), the gas concentration *n* was calculated by the relation *n* = *ε*_*0*_*λφ*⁄*παd*, where *ε*_0_ is the vacuum permittivity, *λ* = 632 nm is the laser wavelength, *α* = 18.52 × 10^–41^ Fm^2^ is the polarizability of the argon gas atoms, and *d* = 17 µm is the reconstruction length of cubic voxels. The tomographic reconstruction of the phase volume profile was calculated using Simultaneous Iterative Reconstruction Technique (SIRT) algorithm^55^. The reconstructed concentration profiles of the nozzles S1 and 2.25 mm nozzle of Arr3 at the backing pressure of 30 bar argon are presented in Fig. 15a,b, respectively. The characterisation has shown that the gas target of the single slit converging–diverging 1.5 mm nozzle S1 has the 0.9 mm-long gas concentration plateau and the ramps with the length of 0.8 mm at each side of the nozzle. The single cylindrical Laval nozzle with the diameter of 2.25 mm has the 1.5 mm-long gas concentration plateau and the ramps with the length of 0.6 mm at each side of the nozzle. The shadowgrams of the single nozzle S1 (c), and array Arr4 in injector geometry (d) are shown in Fig. 15c,d, respectively.

Estimating the dependence of plasma concentration n_p_ on backing pressure, the approach of ideal gases was used. The molecular concentration of ideal gases n_g_ was calculated as *n*_g_ = *N*_A_ *ρ*/*M*, where *N*_A_ is the Avogadro constant, *ρ* is the gas density, and *M* is the molar mass of the gas^56^. At the standard temperature and pressure of *T* = 273.15 K, and *P* = 101.325 kPa, *n*_g_ corresponds to 2.687 × 10^25^ m^−3^. For the calculation of the molecular gas concentration of the same gas under two different sets of the pressure *P*_1,2_, volume *V*_1,2_ and temperature *T*_1,2_, the relation *p*_1_*V*_1_/*T*_1_ = *p*_2_*V*_2_/*T*_2_ was used. The molecular gas concentration increases proportionally to the ratio of actual pressure and temperature. The molecular concentration in the central part of the Arr2 cylindrical nozzle at the backing pressure of 26 bar and *n*_g_ was estimated as 5 × 10^18^ cm^−3^. The same concentration for the S1 nozzle was reached at the backing pressure of 18 bar. Calculating the plasma concentration *n*_p_, it was assumed that helium is fully ionized to the level of He^+2^, and helium plasma concentration *n*_p_ corresponding to the *n*_g_ = 5 × 10^18^ cm^−3^ was estimated as *n*_p_ = 1 × 10^19^ cm^−3^.

The Figs. 3, 4, 5, 6, 7, 8, 9, 10, 11, 12, 13, 14 and 15 and the Fig. S1 in the Supplementary information were visualized using Matplotlib plotting library for the Python programming language^57^.

## Supplementary information

Supplementary file 1

## Data Availability

The authors confirm that all of the data used in this study are available from the corresponding author on reasonable request.

## References

[1] Esarey E, Schroeder CB, Leemans WP (2009). Physics of laser-driven plasma-based electron accelerators. Rev. Mod. Phys..

[2] Corde S (2013). Femtosecond x rays from laser-plasma accelerators. Rev. Mod. Phys..

[3] Albert F, Thomas AGR (2016). Applications of laser wakefield accelerator-based light sources. Plasma Phys. Controlled Fusion.

[4] Sprangle P (2001). Wakefield generation and GeV acceleration in tapered plasma channels. Phys. Rev. E Stat. Nonlinear Soft Matter Phys..

[5] Matlis NH (2016). Transient behavior of a supersonic three-dimensional micronozzle with an intersecting capillary. J. Appl. Phys..

[6] Gallardo González I (2018). Effects of the dopant concentration in laser wakefield and direct laser acceleration of electrons. New J. Phys..

[7] Zeng M, Chen M, Sheng ZM, Mori WB, Zhang J (2014). Self-truncated ionization injection and consequent monoenergetic electron bunches in laser wakefield acceleration. Phys. Plasmas.

[8] Koehler, A. *et al.* Minimizing betatron coupling of energy spread and divergence in laser-wakefield accelerated electron beams. https://arxiv.org/abs/1905.02240 (2019).

[9] Fubiani G, Esarey E, Schroeder CB, Leemans WP (2004). Beat wave injection of electrons into plasma waves using two interfering laser pulses. Phys. Rev. E Stat. Phys. Plasmas Fluids Relat. Interdiscip. Topics.

[10] Hansson M, Aurand B, Ekerfelt H, Persson A, Lundh O (2016). Injection of electrons by colliding laser pulses in a laser wakefield accelerator..

[11] Ta Phuoc K (2008). Betatron radiation from density tailored plasmas. Phys. Plasmas.

[12] Schmid K (2010). Density-transition based electron injector for laser driven wakefield accelerators. Phys. Rev. Spec. Topics Acceler. Beams.

[13] Gonsalves AJ (2011). Tunable laser plasma accelerator based on longitudinal density tailoring. Nat. Phys..

[14] Vargas M (2014). Improvements to laser wakefield accelerated electron beam stability, divergence, and energy spread using three-dimensional printed two-stage gas cell targets. Appl. Phys. Lett..

[15] Zhang Z (2015). Generation of high quality electron beams from a quasi-phase-stable cascaded laser wakefield accelerator with density-tailored plasma segments. New J. Phys..

[16] Hansson M (2016). Localization of ionization-induced trapping in a laser wakefield accelerator using a density down-ramp. Plasma Phys. Controlled Fusion.

[17] Swanson KK (2017). Control of tunable, monoenergetic laser-plasma-accelerated electron beams using a shock-induced density downramp injector. Phys. Rev. Acceler. Beams.

[18] Hansson M (2015). Down-ramp injection and independently controlled acceleration of electrons in a tailored laser wakefield accelerator. Phys. Rev. Spec. Top. Acceler. Beams.

[19] Faure J (2019). A review of recent progress on laser-plasma acceleration at kHz repetition rate. Plasma Phys. Controlled Fusion.

[20] Ferri J (2018). High-Brilliance Betatron γ -Ray Source Powered by Laser-Accelerated Electrons. Phys. Rev. Lett..

[21] Zhu, X.-L. *et al.* Extremely brilliant GeV γ-rays from a two-stage laser-plasma accelerator. *Sci. Adv.* **6**, eaaz7240 (2020). 10.1126/sciadv.aaz7240 PMC7259925 32523994

[22] Stuart BC, Feit MD, Rubenchik AM, Shore BW, Perry MD (1995). Laser-induced damage in dielectrics with nanosecond to subpicosecond pulses. Phys. Rev. Lett..

[23] Butkutė A (2020). Optical damage thresholds of microstructures made by laser three-dimensional nanolithography. Opt. Lett..

[24] Xu SZ (2016). Experimental study on 800 nm femtosecond laser ablation of fused silica in air and vacuum. Nucl. Instrum. Methods Phys. Res., Sect. B.

[25] Mannion PT, Magee J, Coyne E, O’Connor GM, Glynn TJ (2004). The effect of damage accumulation behaviour on ablation thresholds and damage morphology in ultrafast laser micro-machining of common metals in air. Appl. Surf. Sci..

[26] Weber, M. J. *Handbook of Optical Materials*. *Handbook of Optical Materials* (CRC Press, 2018). 10.1201/9781315219615.

[27] Prencipe I (2017). Targets for high repetition rate laser facilities: Needs, challenges and perspectives. High Power Laser Sci. Eng..

[28] Rovige, L. *et al.* Demonstration of stable long-term operation of a kilohertz laser-plasma accelerator. *Phys. Rev. Acceler. Beams* **23**, 093401 (2020).

[29] Tomkus V (2018). High-density gas capillary nozzles manufactured by hybrid 3D laser machining technique from fused silica. Opt. Express.

[30] Tomkus V (2019). Impact of the wall roughness on the quality of micrometric nozzles manufactured from fused silica by different laser processing techniques. Appl. Surf. Sci..

[31] Döpp A (2016). 3D printing of gas jet nozzles for laser-plasma accelerators. Rev. Sci. Instrum..

[32] Schmid K, Veisz L (2012). Supersonic gas jets for laser-plasma experiments. Rev. Sci. Instrum..

[33] Lehe R, Kirchen M, Andriyash IA, Godfrey BB, Vay JL (2016). A spectral, quasi-cylindrical and dispersion-free Particle-In-Cell algorithm. Comput. Phys. Commun..

[34] Kostyukov I, Kiselev S, Pukhov A (2003). X-ray generation in an ion channel. Phys. Plasmas.

[35] Schnell M (2015). Characterization and application of hard X-ray betatron radiation generated by relativistic electrons from a laser-wakefield accelerator. J. Plasma Phys..

[36] Köhler A (2016). Single-shot betatron source size measurement from a laser-wakefield accelerator. Nucl. Instrum. Methods Phys. Res., Sect. A.

[37] OpenCFD Ltd. The Open Source CFD Toolbox, Version v1906. (2019).

[38] Maddox BR, Park HS, Remington BA, McKernan M (2008). Calibration and characterization of single photon counting cameras for short-pulse laser experiments. Rev. Sci. Instrum..

[39] Fourment C (2009). Broadband, high dynamics and high resolution charge coupled device-based spectrometer in dynamic mode for multi-keV repetitive x-ray sources. Rev. Sci. Instrum..

[40] Fourmaux S (2011). Demonstration of the synchrotron-type spectrum of laser-produced Betatron radiation. New J. Phys..

[41] Hussein AE (2019). Laser-wakefield accelerators for high-resolution X-ray imaging of complex microstructures. Sci. Rep..

[42] Kneip S (2010). Bright spatially coherent synchrotron X-rays from a table-top source. Nat. Phys..

[43] Tilikin IN, Shelkovenko TA, Pikuz SA, Hammer DA (2013). Determination of the size of a radiation source by the method of calculation of diffraction patterns. Opt. Spectrosc. (English translation of Optika i Spektroskopiya).

[44] Svendsen K (2018). Optimization of soft X-ray phase-contrast tomography using a laser wakefield accelerator. Opt. Express.

[45] Gureyev TE (2008). Some simple rules for contrast, signal-to-noise and resolution in in-line x-ray phase-contrast imaging. Opt. Express.

[46] Wilkins SW (2014). On the evolution and relative merits of hard X-ray phase-contrast imaging methods. Philos. Trans. R. Soc. A Math. Phys. Eng. Sci..

[47] Rousse A (2004). Production of a keV X-ray beam from synchrotron radiation in relativistic laser-plasma interaction. Phys. Rev. Lett..

[48] Hansson, M. *et al.* Localization of ionization-induced trapping in a laser wakefield accelerator using a density down-ramp. *Plasma Phys. Controll. Fusion ***58**, 055009 (2016).

[49] Buck A (2010). Absolute charge calibration of scintillating screens for relativistic electron detection. Rev. Sci. Instrum..

[50] Kirkpatrick P (1944). Theory and use of ross filters II. Rev. Sci. Instrum..

[51] Gonzalez IG (2018). Development and Applications of a Laser-Wakefield X-ray Source.

[52] Gečys P, Dudutis J, Račiukaitis G (2015). Nanosecond laser processing of soda-lime glass. J. Laser Micro/Nanoeng..

[53] Marcinkevičius A (2001). Femtosecond laser-assisted three-dimensional microfabrication in silica. Opt. Lett..

[54] van Aarle W (2016). Fast and flexible X-ray tomography using the ASTRA toolbox. Opt. Express.

[55] Gregor J, Benson T (2008). Computational analysis and improvement of SIRT. IEEE Trans. Med. Imaging.

[56] Engineering toolbox, https://www.engineeringtoolbox.com/material-properties-t_24.html. (2009).

[57] Matplotlib: Python plotting—Matplotlib 3.3.1 documentation. https://matplotlib.org/.

