# Supplementary material for: Laser wakefield accelerated electron beams and betatron radiation from multijet gas targets
Source: Sci Rep. 2020 Oct 8;10:16807. doi: 10.1038/s41598-020-73805-7 (PMC7545103; doi:10.1038/s41598-020-73805-7)
Supplement: Supplementary file 1 [file 41598_2020_73805_MOESM1_ESM.docx]

Supplementary Information for “Laser Wakefield Accelerated Electron Beams and Betatron Radiation from Multijet Gas Targets”

Vidmantas Tomkus^1*^, Valdas Girdauskas^1,2^ , Juozas Dudutis^1^ , Paulius Gečys^1^ , Valdemar Stankevič^1^ , Gediminas Račiukaitis^1^ , Isabel Gallardo González^3^, Diego Guénot^3^, Jonas Björklund Svensson^3^, Anders Persson^3^ and Olle Lundh^3^

^1^Center for Physical Sciences and Technology, Vilnius, LT-02300, Lithuania

^2^Vytautas Magnus University, Kaunas, LT-44248, Lithuania

^3^Department of Physics, Lund University, Lund, S-221 00 Sweden

* vidmantas.tomkus@ftmc.lt

**Calculation of simulated parameters of betatron radiation**

The number of X-ray photons per shot and the brightness of an electron-generated betatron radiation were evaluated by postprocessing of data of momenta and spatial distribution of FBPIC PIC simulation. The spectral density of synchrotron radiation into the space angle in the direction of the laser radiation at *θ* = 0, generated by one electron at the corresponding critical energy *E_c_* was calculated using following analytical expressions:

where *N*_β_ is the number of oscillations of one electron in the diameter of betatron channel, *E*_r_ is the energy of radiated photons, *ħ* is the reduced Planck constant, *ε_0_* - the vacuum permittivity, *c* - speed of light in vacuum, and *K_2/3_* is the modified Bessel function of the second kind. *E*_c_ in practical units for each of the accelerated electrons was defined using the relation:

where *n_p_* is the plasma concentration in cm^-3^, *r_β_* is the radius of electron oscillations in µm*,* and *γ* is the Lorenz factor of the electron. The cyclic frequency of the electron oscillation is expressed as $\omega_{\beta}=\omega_{p}/\sqrt{2\gamma}$, where *ω_p_* is the cyclic frequency of the plasma. The fundamental wavelength of the radiation generated by the betatron is $\lambda_{r}=\lambda_{\beta}/2\gamma^{2}$, where *λ_β_* is the wavelength of the electron betatron oscillation. The number of electron oscillations in the diameter of the betatron channel was estimated by the relation:

$$N_{\beta}=4.7\times10^{10}\frac{1}{\lambda_{L}[\text{μm}]\sqrt{\gamma n_{p}[\text{cm}^{\text{-3}}]}}\frac{L}{\lambda_{L}},$$

where *L = 2r_β_* and *λ_L_* is the laser wavelength. If $r_{\beta}^{2}/2\gamma_{z}\ll1$, the ratio of the radius of the electron oscillation to the $\sqrt{2\gamma_{z}}$is approximately equal to the ratio of the components of the electron momentum in the transverse and longitudinal directions $r_{\beta}^{2}/2\gamma_{z}\simeq p_{\perp}^{2}/p_{z}^{2}\simeq p_{\perp}^{2}/\gamma_{z}^{2}$, or the ratio of the Lorenz factors in the transverse and longitudinal direction. Based on the relations above, the betatron radius of electron oscillations was calculated as $r_{\beta}=p_{\perp}\sqrt{2/\gamma_{z}}$. The betatron synchrotron strength parameter *K* was estimated using the relation:

$K=\gamma k_{b}r_{\beta}=1.33\times10^{-10}\sqrt{\gamma n_{p}[\text{cm}^{\text{-3}}]}\times r_{\beta}[\text{μm}],$ (4)

where *k_b_* is the electron oscillation wave vector. The angle of the betatron radiation in the oscillation plane was calculated as $\theta_{0.5\perp}=K/\gamma$, and the divergence $\theta_{\perp}=2\theta_{0.5\perp}$. The angle of radiation in perpendicular plane was calculated as $\theta_{0.5s}=1/\gamma$ and $\theta_{s}=2\theta_{0.5s}$. The number of photons *N_phot1_* ~~at~~ ~~FWHM~~ of one electron at FWHM (Full Width Half Maximum) level was calculated by dividing the spectral density over the radiated photon energy *E_r_=ħω_r_*, integrating in the range of energies from *E_min_=*0 eV to *E_max_=*30 keV of sensitivity of X-ray camera and multiplying by stereo angle of *πθ_⊥_θ_s_*/8:

$N_{phot1}=\int_{\theta_{\min}}^{\theta_{\max}} \int_{E_{\min}}^{E_{\max}} \left.\frac{1}{E_{r}}\frac{d^{2}I}{\partial E_{r}\partial\Omega}\right|_{\theta=0}dE_{r}d\Omega=\frac{\pi\theta_{\perp}\theta_{s}}{8}\int_{E_{\min}}^{E_{\max}} \left.\frac{1}{E_{r}}\frac{dI}{dE_{r}}\right|_{\theta=0}dE_{r}=\int_{E_{\min}}^{E_{\max}} \frac{dN_{phot1}}{dE_{r}}dE_{r}.$ (5)

The total number of photons per shot *N_phot_* was estimated by summing the spectra of number of photons generated by electrons with critical energy of *E*_ci_ and integrating in the range of energies from *E_min_=*0 eV to *E_max_=*30 keV:

$N_{phot}=\int_{E_{\min}}^{E_{\max}} \sum_{i} \frac{dN_{phot1}(E_{ci})}{dE_{r}}dE_{r}.$ (6)

The total brightness *Br* of all electrons in photons/s/mrad^2^/mm^2^/0.1% bandwidth units was calculated as:

$Br=\int_{E_{\min}}^{E_{\max}} \sum_{i} \frac{1}{2000\pi r_{\beta i}^{2}\tau}\frac{dN_{phot1}(E_{ci})}{dE_{r}}dE_{r},$ (7)

where *r_βi_* is the radius of electron oscillations expressed in mm, and *τ* is the duration of laser pulse.

**Calculation of experimental parameters of betatron radiation**

The number of photons per shot *N_phot_* and brightness *Br* of the betatron X-ray source at FWHM level was defined by postprocessing the data of X-ray images. The number of photons per shot *N_phot_* was calculated as:

$N_{phot}=N_{photC}N_{c}$ (8)

where *N_photC_* is the number of photons per count and *N_c_* is the number of counts.


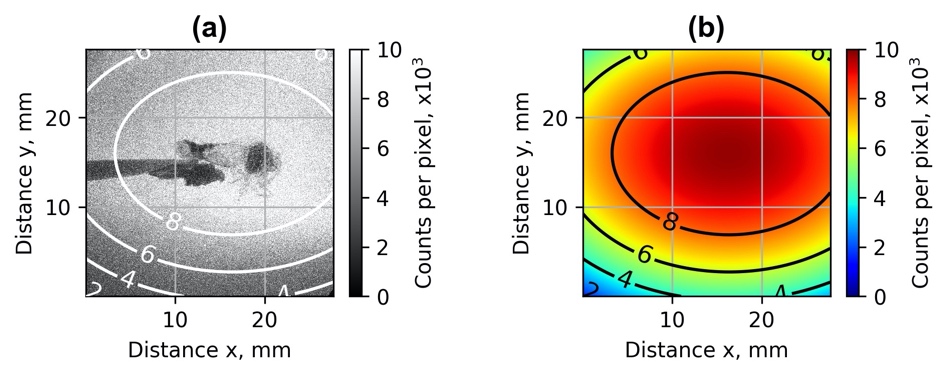


**Figure 1.** Evaluation of standard deviation of normal Gaussian distribution of radiation of X-ray source.

The number of counts *N_c_* was estimated by integrating the function of spatial distribution of counts per pixel *C(x_px_, y_px_)* over the coordinates *x_px_* and *y_px_* expressed in pixel number:

$N_{c}=\int_{-\infty}^{+\infty} C(x_{px},y_{px})dx_{px}dy_{px}.$ (9)

The function *C(x_px_, y_px_)* was assumed to consist of two parts: Gaussian distribution of X-ray radiation and background part *C_bg_*:

$C(x_{px},y_{px})=C_{gd}e^{-\frac{(x_{pxr}-x_{pxc})^{2}}{2\sigma_{x}^{2}}+\frac{(y_{pxr}-y_{pxc})^{2}}{2\sigma_{y}^{2}}}+C_{bg},$ (10)

where *C_gd_* is the amplitude of Gaussian part of counts per pixel, *x_rpx_* and *y_rpx_* are coordinates of pixels tilted by angle *θ_gd_*: $x_{pxr}=x_{px}\cos(\theta_{gd})-y_{px}\sin(\theta_{gd})$ and $y_{pxr}=x_{px}\sin(\theta_{gd})+y_{px}\cos(\theta_{gd})$, *x_pxc_* and *y_pxc_* are the center coordinates of Gaussian distribution, and *σ_x_* and *σ_y_* are standard deviation of Gaussian distribution of X-ray counts per pixel in x and y-direction (Fig. 1). *C_bg_* was estimated by averaging of counts per pixel of the first and last column as well as upper and lower row of X-ray image. The initial parameters of Gaussian distribution $x_{pxc}^{0},\text{ }y_{pxc}^{0},\text{ }\sigma_{x}^{0},\text{ }\sigma_{y}^{0},\text{ }\theta_{gd}^{0},\text{ }C_{bg}^{0}$ were calculated using following relations:

$x_{pxc}^{0},y_{pxc}^{0}=\frac{\sum_{i}^{n} X,Y_{pxi}(C_{i}-C_{bg})}{\sum_{i}^{n} (C_{i}-C_{bg})},$ (11)

(12)

$M_{xx,yy}==\frac{\sum_{i}^{n} (X,Y_{pxi}-x,y_{pxc})^{2}(C_{i}-C_{bg})}{\sum_{i}^{n} (C_{i}-C_{bg})},$ (13)

$M_{xy}=\frac{\sum_{i}^{n} (X_{pxi}-x_{pxc})(Y_{pxi}-y_{pxc})(C_{i}-C_{bg})}{\sum_{i}^{n} (C_{i}-C_{bg})},$ (14)

$\theta_{gd}^{0}=0.5arctg\left(\frac{2M_{xy}}{M_{xx}-M_{yy}}\right),$ (15)

where *X_pxi_* and *Y_pxi_* are the coordinate grids of rows and columns of X-ray image, *C_i_* – counts per pixel of *i*-th pixel, *C_xci_* and *C_yci_* are correspondingly the row and column of counts per pixel at central coordinates of Gaussian distribution of *x_pxc_* and *y_pxc_*, and *M_xx_*, *M_yy_* and *M_xy_* are the momenta of Gaussian distribution used for calculation of tilting angle *θ_gd_*. The initial parameters of Gaussian distribution $x_{_{pxc}}^{0},\text{ }y_{pxc}^{0},\text{ }\sigma_{x}^{0},\text{ }\sigma_{y}^{0},\text{ }\theta_{gd}^{0},\text{ }C_{bg}^{0}$ were optimized by calculating the minimum of squared difference between the measured X-ray image data and the best fit of Gaussian distribution function:

$f(C_{gd},x_{pxc},y_{pxc},\sigma_{x},\sigma_{y},\theta_{gd},C_{bg})=C_{gd}e^{-\frac{(x_{pxr}-x_{pxc})^{2}}{2\sigma_{x}^{2}}+\frac{(y_{pxr}-y_{pxc})^{2}}{2\sigma_{y}^{2}}}+C_{bg}.$ (16)

The spatial distribution of illumination exceeded the size of X-ray sensors, therefore, afterwards only the Gaussian part of the X-ray radiation was considered, and the total number of counts *N_c_* was estimated assuming:

$N_{c}=\int_{-\infty}^{+\infty} C_{gd}e^{-\frac{(x_{rpx}-x_{cpx})^{2}}{2\sigma_{x}^{2}}+\frac{(y_{rpx}-y_{cpx})^{2}}{2\sigma_{y}^{2}}}dx_{px}dy_{py}=C_{gd}2\pi\sigma_{x}\sigma_{y}.$ (17)

The number of counts at FWHM level was calculated as:

$N_{cFWHM}=C_{gd}2\ln2\frac{\pi\sigma_{x}\sigma_{y}}{2}.$ (18)

The number of photons per count *N_photC_* was estimated as:

$N_{photC}=\alpha_{c}S(E,E_{c}),$ (19)

where *α_c_* = 11.9 eV/counts - the X-ray detector sensitivity calibration constant. $S(E,E_{c})=S_{1}/S_{eff}$is the normalized synchrotron-like spectrum calculated in energy range from *E_min_=*0 eV to *E_max_=*30 keV as:

$S_{1}(E,E_{c})=\int_{E_{\min}}^{E_{\max}} \left(\frac{E}{E_{c}}\right)^{2}K_{\frac{2}{3}}^{2}\left(\frac{E}{2E_{c}}\right)dE,$ (20)

$S_{eff}(E,E_{c})=\int_{E_{\min}}^{E_{\max}} \left(\frac{E}{E_{c}}\right)^{2}K_{\frac{2}{3}}^{2}\left(\frac{E}{2E_{c}}\right)T_{total}(E)\cdot Q_{e}(E)EdE,$ (21)

where *E_c_* is the critical betatron radiation energy measured by Ross filters, *T_total_*(*E*) is the pass factor of all materials placed between the X-ray source and X-ray camera, *Q*_e_(*E*) is the quantum yield of the Andor iKon-L SO BR-DD camera. In the calculations, the attenuation of 3 µm aluminum, 250 µm Kapton, 250 µm Beryllium and 120 mm of air was evaluated.

The halves of the divergence in mrad in x and y-direction of radiation *θ_0.5x_, θ_0.5y_* were defined estimating the standard deviation *σ_x_* and *σ_y_* of normal Gaussian distribution of X-ray counts per pixel in x and y-direction as:

$\theta_{0.5x,y}=1000arctg\left(\sqrt{2\ln2}\frac{\sigma_{x,y}l_{px}}{l_{sd}}\right),$ (22)

where *l_px_* = 13.5 µm is the pixel size and *l_s_*_d_ = 730 mm is the distance between the source and X-ray camera detector. The averaged divergence of betatron radiation was calculated as *θ_⊥β_ = θ_0.5y_ + θ_0.5x_*. The brightness of the source in units of photons/s/mm^2^/mrad^2^/0.1%BW was calculated as:

$Br=\frac{N_{phot}}{1000\pi\theta_{0.5x}\theta_{0.5y}\pi r_{\beta}^{2}\tau},$ (23)

where *r_β_* – measured radius of betatron source of 5 µm in mm, and *τ* is the duration of the laser pulse.
